# *Wedelia trilobata*-derived biochar mitigates chromium toxicity and improves physiological performance in hydroponically grown Chinese cabbage

**DOI:** 10.3389/fpls.2025.1624352

**Published:** 2025-09-09

**Authors:** Fengyue Qin, Weidong Li, Menglu Dong, Shuangqi Yue, Guojie Weng, Mingxuan Wang, Xinyu Shan, Waqas Ahmed, Jiechang Weng, Sajid Mehmood

**Affiliations:** ^1^ Center for Eco-Environment Restoration of Hainan Province, School of Ecology, Hainan University, Haikou, China; ^2^ School of Topical Agriculture and Foresty, Hainan University, Haikou, China; ^3^ Hainan Provincial Ecological and Environmental Monitoring Center, Haikou, China

**Keywords:** chromium stress, biochar, *Wedelia trilobata*, Chinese cabbage (*Brassica rapa*), Chinese cabbage

## Abstract

**Introduction:**

Ensuring future agricultural sustainability requires innovative solutions to alleviate abiotic stress caused by heavy metal(loid) contamination. Chromium (Cr) toxicity is a major abiotic stressor that threatens leafy vegetable productivity and food safety.

**Methods:**

This study investigates the potential of Wedelia trilobata-derived biochar (WBC= 0, 0.1, 0.5, 1, and 3 g L-1) to mitigate Cr-induced abiotic stress (Cr = 50 mg/L, 7 days) in hydroponically grown Chinese cabbage (Brassica rapa). WBC was synthesized, characterized, and applied at varying concentrations in a flow-through hydroponic system.[Results] Results showed that WBC exhibited a strong chromium adsorption capacity. At an application rate of 3 g/L (T5), chromium accumulation in plant shoots and roots was significantly reduced by 97.12% and 97.15%, respectively, compared to the pure chromium treatment group. In the same treatment (T5), the total chlorophyll and carotenoid contents in plant shoots increased by 128.47% and 183.33%, respectively. Additionally, the malondialdehyde (MDA) content decreased by 29.66% in roots and 15.98% in shoots. Hydrogen peroxide (H₂O₂) levels were reduced by 33.95% in roots and 59.22% in shoots. Proline content also declined by 62.85% in roots and 79.78% in shoots. Conversely, the soluble protein content increased by 17.43% in roots and 28.13% in shoots, while soluble sugar levels rose by 78.09% in roots and 502.35% in shoots. At a lower application rate of 1 g/L (T4), plant root and shoot dry weights increased by 92.39% and 71.57%, respectively. Root length and shoot length also improved by 9.82% and 24.93%. Moreover, calcium and magnesium contents in plant shoots significantly increased by 478.99% and 97.86%, respectively.

**Discussion:**

WBC application enhanced plant stress tolerance by boosting photosynthetic pigments and antioxidant enzyme activities including superoxide dismutase (SOD), peroxidase (POD), and catalase (CAT) while reducing oxidative stress indicators such as proline (PRO). Furthermore, WBC improved macro-nutrient uptake, notably increasing levels of N, P, K, Ca, and Mg. These findings highlight WBC as an effective amendment for alleviating heavy metal-induced abiotic stress, promoting healthier plant growth, and enhancing nutrient assimilation. This study offers valuable insights into biochar-mediated stress mitigation, with promising implications for sustainable agriculture and environmental remediation.

## Graphical Abstract

The abstract should ideally be structured according to the IMRaD format (Introduction, Methods, Results and Discussion). Provide a structured abstract if possible. If your article has been copyedited by us, please provide the updated abstract based on this version.

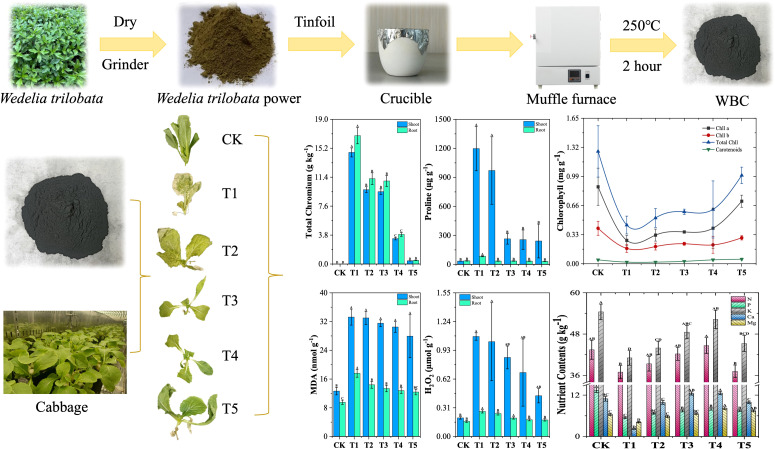

## 1 Introduction

Buildup of heavy metals (HMs) due to increase in population, industrialization and urbanization has led to environmental pollution (Kapoor et al., 2022), including chromium (Cr), pose a significant threat to ecosystems and human health as they enter the food chain through various sources (Ali et al., 2023; Das et al., 2023; Saravanan et al., 2024). Cr is extensively utilized in industries such as tanning, textiles, and electroplating, which contribute to economic development but also result in severe environmental pollution (Qi et al., 2023). Additionally, Cr mining and improper disposal produce large quantities of Cr-rich waste, leading to air and water contamination (Sharma et al., 2022). Prolonged exposure to Cr can induce a range of pathological effects in humans, impacting vital organs such as the liver, kidneys, and respiratory and reproductive systems (Hossini et al., 2022). In plants, Cr toxicity manifests as delayed seed germination, root damage, growth inhibition, and reduced photosynthetic capacity (Srivastava et al., 2021). Furthermore, hexavalent Cr (Cr (VI)) adversely affects crop growth and quality by disrupting the activity of key antioxidant enzymes, including catalase, peroxidase, and superoxide dismutase (Singh et al., 2022). The imperative to reduce Cr contamination in the environment, particularly in leafy vegetables, is of paramount importance. Due to their direct consumption by humans, leafy vegetables are a critical pathway for Cr exposure, posing significant health risks. Various treatments have been investigated to remove Cr from soil and water; among these, biochar, a carbon-rich material, has emerged as the most efficacious adsorbent (Xia et al., 2020). Biochar, due to its large specific surface area, stable structure, high cation exchange capacity, and tunable physicochemical properties, is highly suitable for environmental applications (Aftab et al., 2022; Sun et al., 2022). While the majority of biochar research has focused on its production from crop residues, limited attention has been given to the utilization of invasive wild herbs, which compete with essential crops and reduce agricultural productivity (Feng et al., 2021).

Many researchers indicated that biochar demonstrates efficacy in immobilizing Cr, thereby reducing its bioavailability and mitigating Cr-induced stress on plants. Hashem et al. (2020) reported that water hyacinth biochar achieved up to 99% removal of Cr from tannery wastewater within 15 minutes. Shrimp-Waste-Derived Biochar increased the metal toxicity tolerance of wastewater-irrigated Chenopodium quinoa and promoted plant growth, as well as significantly reduced Pb and Cd concentrations in plant leaves and reduced toxic metal levels in plant seeds (Mousa et al., 2022). Calcium-rich shrimp waste biochar application increased the soil quality and nutrient uptake by pearl millet plants (Abo-Elyousr et al., 2022). These findings underscore the considerable potential of biochar derived from diverse feedstocks for environmental remediation.

Chinese cabbage (*Brassica rapa* L.) is a crucial leafy vegetable in China, Korea, and other East Asian countries, occupying a central role in consumer markets (Li et al., 2021; Gao et al., 2022). It is extensively cultivated and consumed on a daily basis, both as an independent vegetable and as a component in various dishes, rendering its quality highly significant to the population (Nie et al., 2021). Beyond its culinary importance, Chinese cabbage possesses economic and nutritional value. However, it exhibits high susceptibility to heavy metal contamination, particularly Cr, which can compromise its quality and pose severe risks to human health (Lu et al., 2021; Ahmed et al., 2022). These risks underscore the importance of addressing heavy metal contamination to ensure the safe consumption of Chinese cabbage. Effective control measures are essential to mitigate Cr contamination, thereby maintaining food safety and safeguarding public health.

*Wedelia trilobata*, commonly known as South American Wedelia (*Wedelia trilobata* (L.) Hitchc.), poses a threat to local biodiversity by outcompeting native plants and has been listed as one of the 100 most invasive alien species in the world (Jiang et al., 2024). Furthermore, it is considered an invasive plant in China due to its vigorous spread across various provinces, including Guangxi, Hunan, Fujian, and Guangdong. The species exhibits a well-developed root system, rapid growth, high nutrient absorption capability, and strong antioxidant defense system even at low nutrient availability (Khan et al., 2023). Consequently, the high natural production of *Wedelia trilobata* renders it a suitable candidate for biochar production. Horticultural waste biochar significantly increased the yield of lettuce (*Lactuca sativa* L.) and the concentration of essential mineral nutrients in soil-based systems; In addition, in soilless systems, the extensive use of horticultural waste biochar to replace peat moss has reduced the concentration of heavy metals in lettuce, except for Cr (Yang et al., 2025). To the best of our knowledge, this is the first systematic investigation evaluating the impact of varying concentrations of *Wedelia trilobata*-derived biochar on the growth performance and chromium (Cr) uptake of Chinese cabbage in a hydroponic system. This study uniquely explores the dual role of *W. trilobata* biochar in recycling invasive plant biomass and enhancing plant tolerance to heavy metal stress under controlled hydroponic conditions. In the present study, the potential of *Wedelia trilobata*-biochar to mitigate Cr toxicity and enhance the growth performance of Chinese cabbage was investigated. The research focused on three key objectives: (1) evaluating the efficacy of different concentrations of WBC (0, 0.1, 0.5, 1, and 3 g L^-1^) in reducing Cr bioavailability and improving plant growth through hydroponic trials; (2) assessing the morpho-physiological and biochemical responses of plants to Cr stress, with emphasis on parameters such as photosynthetic pigment levels, antioxidant enzyme activity, and nutrient uptake; and (3) comprehensively analyzing the ability of plants to tolerate and mitigate Cr stress under varying WBC treatments. This approach provides a detailed understanding of the interactions between WBC application and plant response mechanisms under Cr stress conditions. The biochar doses applied in this study were selected based on previously published research, where they demonstrated significant potential for effective remediation (Peng et al., 2021; Yang et al., 2022).

## 2 Materials and methods

### 2.1 Biochar preparation and characterization

To prepare the biochar, *Wedelia trilobata* was collected from roadsides near Hainan University, Haikou, Hainan, China. The collected material was manually cleaned to remove visible dust and washed thoroughly with ultrapure water to eliminate residual soil particles. The cleaned plants were then dried in an oven at 60°C for 24 hours until completely dehydrated. Once dried, the plants were cooled to room temperature and finely ground using a grinder to obtain a homogeneous powder, which was stored in clean Ziplock bags for later use. A portion of the powder was wrapped in tinfoil and placed in a crucible, which was then heated in a muffle furnace at 250°C for 2 hours (Yang et al., 2021). One of the methods for preparing biochar torrefaction is value-added at lower temperatures (200-300°C) and the temperature range belongs to the category of low-temperature waste heat (<300°C) (Lin et al., 2023). Besides, low-temperature waste heat recovery could be a potential energy source, and its development belongs to a relatively energy-saving technology (Chen et al., 2022; Lin et al., 2023). After heating, the crucible was left to cool down gradually to room temperature over 24 hours. Once cooled, the charred material was removed, reground to ensure uniformity, and stored in clean, sealed bags for further analysis.

The surface morphology of the prepared *Wedelia trilobata* biochar was examined using scanning electron microscopy (SEM, Verios G4 UC, Thermo Fisher (FEI), Czech Republic). Physical phase analysis and crystallinity determination were carried out using X-ray diffraction (XRD, Smart Lab, Rigaku, Japan). The chemical structure and functional groups were analyzed through Fourier transform infrared spectroscopy (FTIR, Thermo Nicolet iS50, Thermo Scientific, USA). Qualitative and quantitative elemental analyses, including elemental distribution and species content (with permissible relative error), were performed using energy-dispersive spectroscopy (EDS) integrated with SEM (Verios G4 UC, Thermo Fisher (FEI), Czech Republic). The specific surface area and porosity were analyzed using nitrogen adsorption-desorption isotherms at 77 K on a BET analyzer.

### 2.2 Cultivation experiment

Cabbage seeds (*Beijing No. 3, Brassica rapa* L. ssp. pekinensis) were sourced from the Hebei Qingxian Vegetable Seed Breeding Base (http://www.xingyunzhongye.com). Prior to planting, the seeds were washed with DI water and subjected to a screening process to remove floating or damaged seeds. The selected seeds were surface sterilized by soaking in a 30% hydrogen peroxide solution for 15 minutes, followed by thorough rinsing three times with ultrapure DI water to ensure sterility (Yin et al., 2024). After rinsing, the seeds were placed on clean cheesecloth in plastic trays to maintain adequate humidity for germination. The trays were then incubated in a climate-controlled growth chamber set at 25/20°C (day/night temperature), with a relative humidity of 70% and a 14/10-hour light/dark cycle (Yin et al., 2024). Germination proceeded for 10 days, during that time the seeds were monitored to ensure proper growth conditions. Healthy and uniform seedlings were selected and transplanted into six-hole hydroponic boxes with a hole diameter of 20 mm and a total volume of 1 L, providing an optimal setup for cabbage cultivation.

The cabbage seedlings were carefully placed into the designated holes in the hydroponic boxes, ensuring that the roots were fully immersed in the Hoagland’s nutrient solution. To stabilize the plants and prevent floating or tilting, planting cotton was used to secure the seedlings. The hydroponic boxes with the plants were then placed in a climate-controlled incubator. The nutrient solution levels were monitored regularly to ensure that the roots remained submerged. Hoagland’s nutrient solution was replaced every 3-4 days to meet the growth requirements of the plants. Poorly growing plants were removed promptly to maintain uniformity, and plant health was monitored throughout the experiment. After ten days, cabbage seedlings with consistent growth were selected for further experimentation.

### 2.3 Experimental setup

The experiment was designed with one control group (CK) and five treatment groups (T1, T2, T3, T4, and T5), each replicated three times. The experimental setup was as follows: Control group (CK): Hoagland’s nutrient solution (prepared by adding Hoagland’s reagent to ultrapure water according to standard guidelines). Pre-screened cabbage seedlings were transplanted into the solution, and the containers were labelled as (CK (Cr = 0 mg/L)) to indicate no chromium or biochar was added. T1: Hoagland’s nutrient solution was mixed with a Cr solution to achieve a final Cr concentration of 50 mg/L. The solution was stirred thoroughly, and pre-screened cabbage seedlings were transplanted. Containers were labelled as [T1 (Cr = 50 mg/L, BC = 0 g/L)]. T2: A mixture of chromium solution and Hoagland’s nutrient solution was prepared as in T1. *Wedelia trilobata* biochar was added to achieve a final concentration of 0.1 g/L. The solution was mixed well, and pre-screened cabbage seedlings were transplanted. Containers were labelled as [T2 (Cr = 50 mg/L, BC = 0.1 g/L)]. T3, T4, and T5: Following the same procedure as T2, *Wedelia trilobata* biochar concentrations were adjusted to 0.5 g/L, 1 g/L, and 3 g/L, respectively, with containers labelled as [T3 (Cr = 50 mg/L, BC = 0.5 g/L); T4 (Cr = 50 mg/L, BC = 1 g/L); and T5 (Cr = 50 mg/L, BC = 3 g/L)].

Biochar has demonstrated potential to enhance plant growth and nutrient uptake in hydroponic systems when its distribution is properly managed (Awad et al., 2017). One challenge is sedimentation, which can lead to non-uniform exposure of roots to biochar’s beneficial properties. To mitigate this, we adopted manual stirring as a practical strategy: each container was gently stirred twice daily for approximately 10 minutes, ensuring a uniform suspension and minimizing particle settling throughout the experimental period. This approach is consistent with prior studies suggesting manual agitation as an effective technique for maintaining homogeneous particle distribution in liquid systems (Meng et al., 2021). The experiment was conducted under controlled conditions in a climatic incubator, matching the environmental parameters used during cabbage cultivation: 25/20°C (day/night), 70% relative humidity, and a 14/10-hour light/dark cycle (Yin et al., 2024). This setup ensured consistent environmental conditions across all groups for reliable comparisons.

### 2.4 Analysis

At the conclusion of the experiment, plants were thoroughly rinsed with ultrapure deionized (DI) water to remove any residual particulate matter. The plant surfaces were then blotted dry. Key growth parameters, including total plant biomass, root and shoot lengths, and root and shoot biomass, were measured and recorded. In addition, the pH and electrical conductivity (EC) of the nutrient solution in each hydroponic container were assessed.

To quantify chromium (Cr) concentrations in the plant roots and leaves, the procedure outlined in the GBT5009.123-2014 National Standard for Food Safety: Determination of Chromium in Foodstuffs by Atomic Absorption Graphite Furnace Method was followed. Total nitrogen content in the plant tissues was determined using an automatic nitrogen determination instrument. Total phosphorus and potassium concentrations were quantified using the methodology described by Bao Shidan in the third edition of Soil Agrochemical Analysis (China Agriculture Press). Plant samples underwent acid digestion with a mixture of strong acids, followed by the determination of calcium and magnesium content via atomic absorption spectroscopy.

Biochemical parameters were extensively analyzed in both shoot and root tissues, including malondialdehyde (MDA), hydrogen peroxide (H_2_O_2_), superoxide dismutase (SOD), catalase (CAT), peroxidase (POD), proline, soluble proteins, and soluble sugars. Photosynthetic pigments, such as chlorophyll a, chlorophyll b, total chlorophyll, and carotenoids, were also quantified. The following assay kits were employed for these determinations: SOD activity was measured using the Superoxide Dismutase-WST-8 Method Activity Assay Kit (G0101W); CAT activity was assessed using the Catalase Activity Kit (G0105W); POD activity was measured with the Catalase Kit (G0107W); MDA content was quantified using the Malondialdehyde Content Kit (G0109W); soluble sugar content was determined with the Soluble Sugar Content Kit (G0501W); proline content was quantified using the Proline Content Assay Kit (G0111W); soluble protein content was analyzed with the Protein Content Assay Kit by Coomassie Brilliant Blue Method (G0417W); chlorophyll content was measured using the Chlorophyll Content Assay Kit (G0601W); and H_2_O_2_ content was quantified with the Hydrogen Peroxide Content Assay Kit (G0168W). These comprehensive analyses were performed to gain a detailed understanding of the effects of varying concentrations of *Wedelia trilobata* biochar on the growth and biochemical responses of cabbage plants exposed to chromium-contaminated nutrient solutions. This thorough evaluation aims to elucidate the potential of biochar to mitigate chromium-induced stress in hydroponic cultivation systems.

### 2.5 Data collection and statistical analysis

In this study, robust statistical methodologies were implemented to ensure the precision and reproducibility of the findings. Analysis of variance (ANOVA) was performed using IBM SPSS Statistics 23 (NY, USA) to compute the mean and standard deviation (± SD) of the dataset. To assess the influence of biochar application, the least significant difference (LSD) test was utilized, with a significance threshold set at p ≤ 0.05. Furthermore, multiple comparisons across treatment groups were conducted using Tukey’s Honestly Significant Difference (HSD) test, establishing a significance level of p < 0.05. Data visualization and graphical representation were executed using Origin 2019b (9.65) (Origin Lab Corporation, Northampton, USA). These analytical techniques facilitated a comprehensive evaluation of treatment differences, offering a scientifically rigorous foundation for assessing the effects of varying biochar concentrations on cabbage growth under chromium (Cr) stress.

## 3 Results and discussion

### 3.1 Characterization of biochar

**Figure 1:**
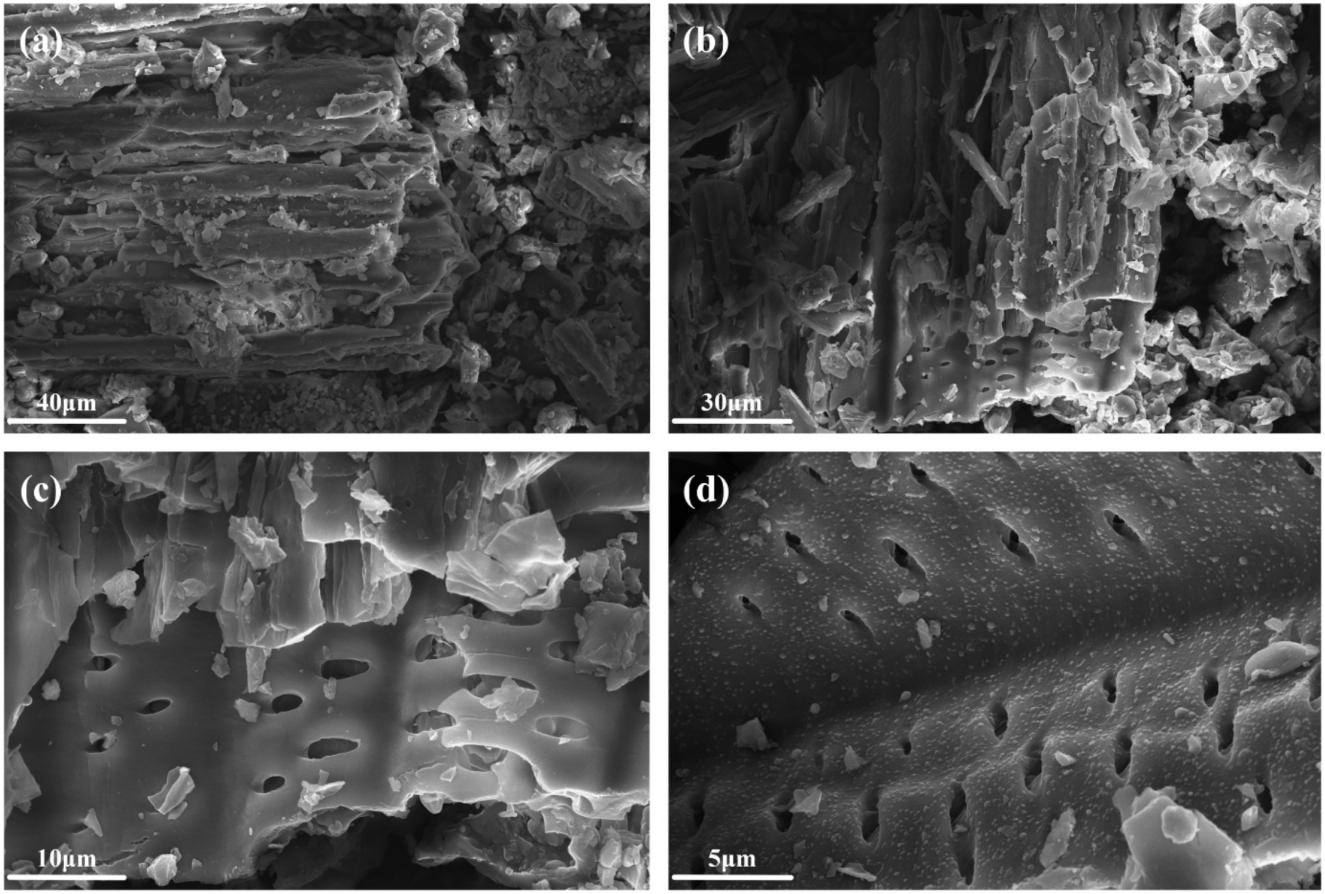
Scanning Electron Microscopy (SEM) images of *Wedelia trilobata*-derived biochar (WBC) at different magnifications. **(a)** At 40 µm and **(b)** 30 µm scales, the layered and fractured architecture is visible, likely resulting from thermal decomposition during pyrolysis. **(c)** At 10 µm magnification, abundant irregular pores are evident, enhancing surface area for adsorption. **(d)** At 5 µm magnification, slit-shaped and elongated pores are observed, which facilitate water retention, nutrient exchange, and potential heavy metal immobilization in hydroponic systems.

**Figure 1** presents scanning electron microscope (SEM) images of *Wedelia trilobata* biochar, highlighting its microstructure and surface morphology. After the pyrolysis process at 250 °C, it can be seen that the fiber structure of the plant powder has undergone obvious changes, resulting in obvious fragmentation, including cracks, collapses, surface roughness, and the formation of a clear pore structure (**Figure 1**). Slow pyrolysis results in the release of volatile organic compounds, hemicellulose, and lignin, as well as shrinkage, melting, and cracking, thereby increasing the porosity of the material (Batista et al., 2018). At a scale of 40 μm (**Figure 1a**), the biochar’s outer contour exhibits a clear, typical tubular pore structure with rougher pores, and the cavity structure of the plant conduits is prominently visible, reflecting its porous characteristics. Upon magnification to 30 μm (**Figure 1b**), 10 μm (**Figure 1c**), and 5 μm (**Figure 1d**), the pores progressively become more regular and denser. The walls of the tubular structures thin out, and regularly arranged small pores appear on the inner surfaces of the tubes. Additionally, multi-diameter pores emerge as the pyrolysis progresses. These observations suggest that the microstructure of *Wedelia trilobata* biochar evolved during the pyrolysis process, resulting in the formation of a distinctive porous structure feature. Due to the presence of a highly porous structure, *Wedelia trilobata* biochar may exhibit a greater affinity for heavy metals (Li et al., 2017).

Scanning Electron Microscopy combined with Energy Dispersive X-ray spectroscopy (SEM-EDX) is a powerful technique for investigating both the morphological and chemical characteristics of materials (Allegretta et al., 2022). **Supplementary Figure S1** illustrates the results of SEM-EDX analysis for *Wedelia trilobata* biochar. (**Supplementary Figure S1a**) displays the overall elemental profile spectra corresponding to the localized area in (**Supplementary Figure S1b**), which provides a detailed view of the biochar sample’s surface. (**Supplementary Figures S1c, d**) depict the spatial distribution of carbon (C) in yellow and oxygen (O) in green within the analytical scanning range. The total elemental distribution map at a scale of 100 μm reveals the presence of carbon (C), oxygen (O), and aluminum (Al) in *Wedelia trilobata* biochar. Quantitatively, the weight percentages of C and O are 67.0% and 32.4%, respectively, at a dissociation cross-section (σ) of 1.0, while the weight percentage of Al is measured at 0.5% with a dissociation cross-section (σ) of 0.2. In general, the C content of biochar is inversely proportional to the biochar yield, that is, the higher the C content, the lower the biochar yield (Mahimairaja and Shenbagavalli, 2012). At the same time, the production of biochar by pyrolysis is a carbonization process in which the carbon content increases with temperature while the oxygen and hydrogen content decreases (Angin et al., 2013). Carbon is the primary element in condensed aromatic structures, which dominate the organic phase of biochar; while O is the key element in many polar organic functional groups on biochar surfaces, which influence biochar reactivity (Bakshi et al., 2020).

**Figure 2:**
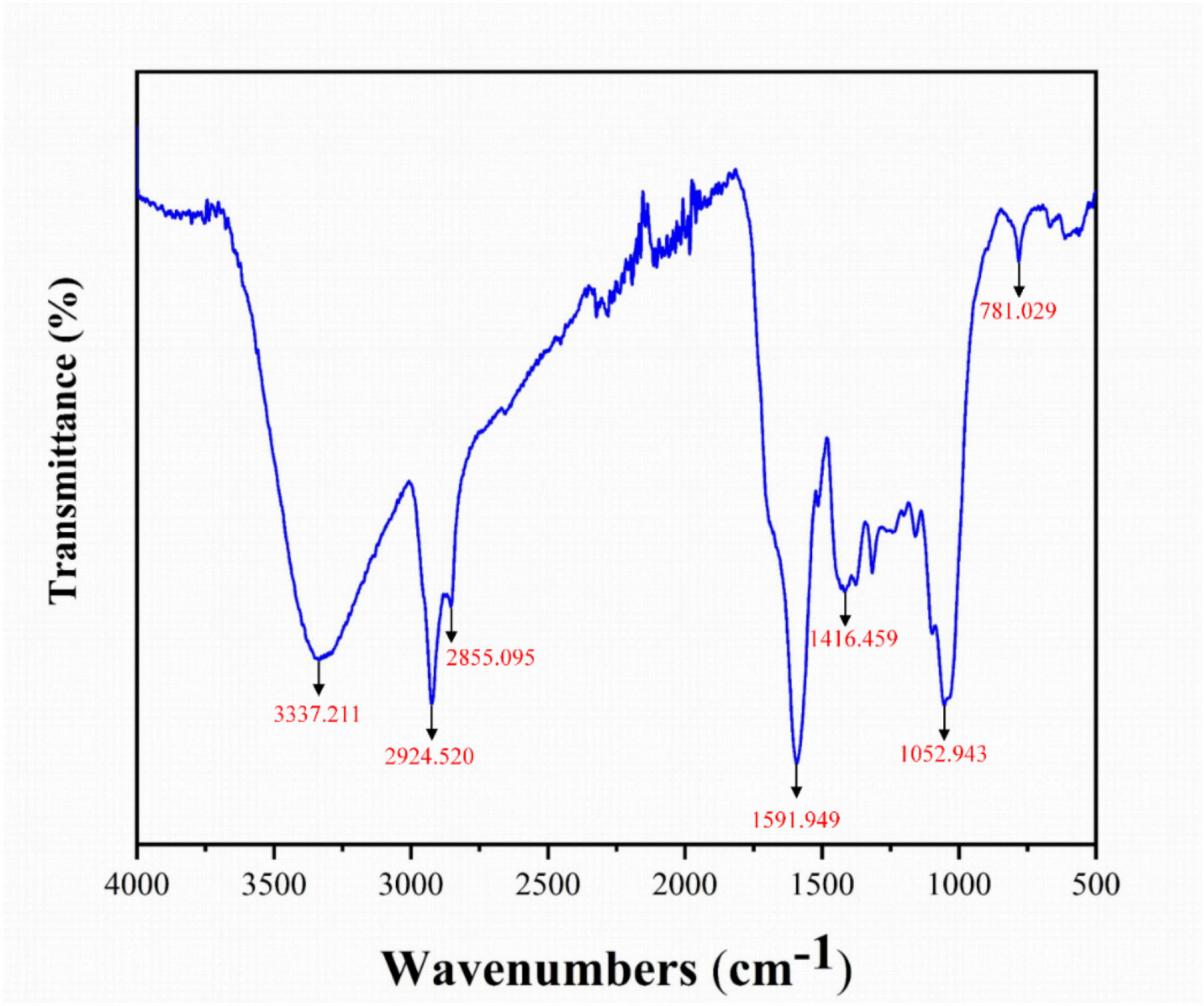
Fourier Transform Infrared (FTIR) spectrum of *Wedelia trilobata*-derived biochar (WBC), showing key functional groups responsible for surface reactivity. The broad band at 3337 cm^-1^ corresponds to O–H stretching vibrations of hydroxyl groups (alcohols or phenols), while peaks at 2924 and 2855 cm^-1^ are attributed to aliphatic C–H stretching. The peak at 1591 cm^-1^ indicates aromatic C=C stretching, and the band at 1416 cm^-1^ corresponds to O–H bending of carboxylic acids. The absorption at 1052 cm^-1^ is associated with C–O stretching in alcohols, phenols, or ethers, and the peak at 781 cm^-1^ indicates aromatic C–H bending.

Fourier Transform Infrared Spectroscopy (FTIR) is an effective technique for analyzing functional groups in biochar, with spectra recorded in the range of 500-4000 cm^-1^. As shown in **Figure 2**, the broad absorption peak at 3337.211 cm^-1^ corresponds to O-H stretching vibrations, indicating the presence of hydroxyl groups derived from glucose molecules in cellulose and hemicellulose, as well as phenolic functional groups in lignin (Chen et al., 2024; Pasumarthi et al., 2024). Peaks at 2924.520 cm^-1^ and 2855.095 cm^-1^ are associated with symmetric C-H stretching vibrations, revealing the presence of saturated hydrocarbon chains and aliphatic hydrocarbons, respectively (Gu et al., 2021; Pasumarthi et al., 2024). The peak at 1591.949 cm^-1^ reflects the stretching vibrations of C=C double bonds, indicating the presence of aromatic structures. As shown in **Figure 2**, the peak at 1416.459 cm^-1^ is linked to C=N stretching vibrations, which may correspond to primary amides, while the peak at 1052.943 cm^-1^ is associated with C=O stretching vibrations, typically found in alcohols, ethers, or esters (Wang et al., 2021). In addition, the peak at 781.029 cm^-1^ indicates the presence of aromatic compounds (Dalavi and Patil, 2016). These diverse functional groups, including hydroxyl, aliphatic, aromatic, and oxygen-containing groups, provide a strong basis for the adsorption properties of *Wedelia trilobata* biochar.

**Figure 3:**
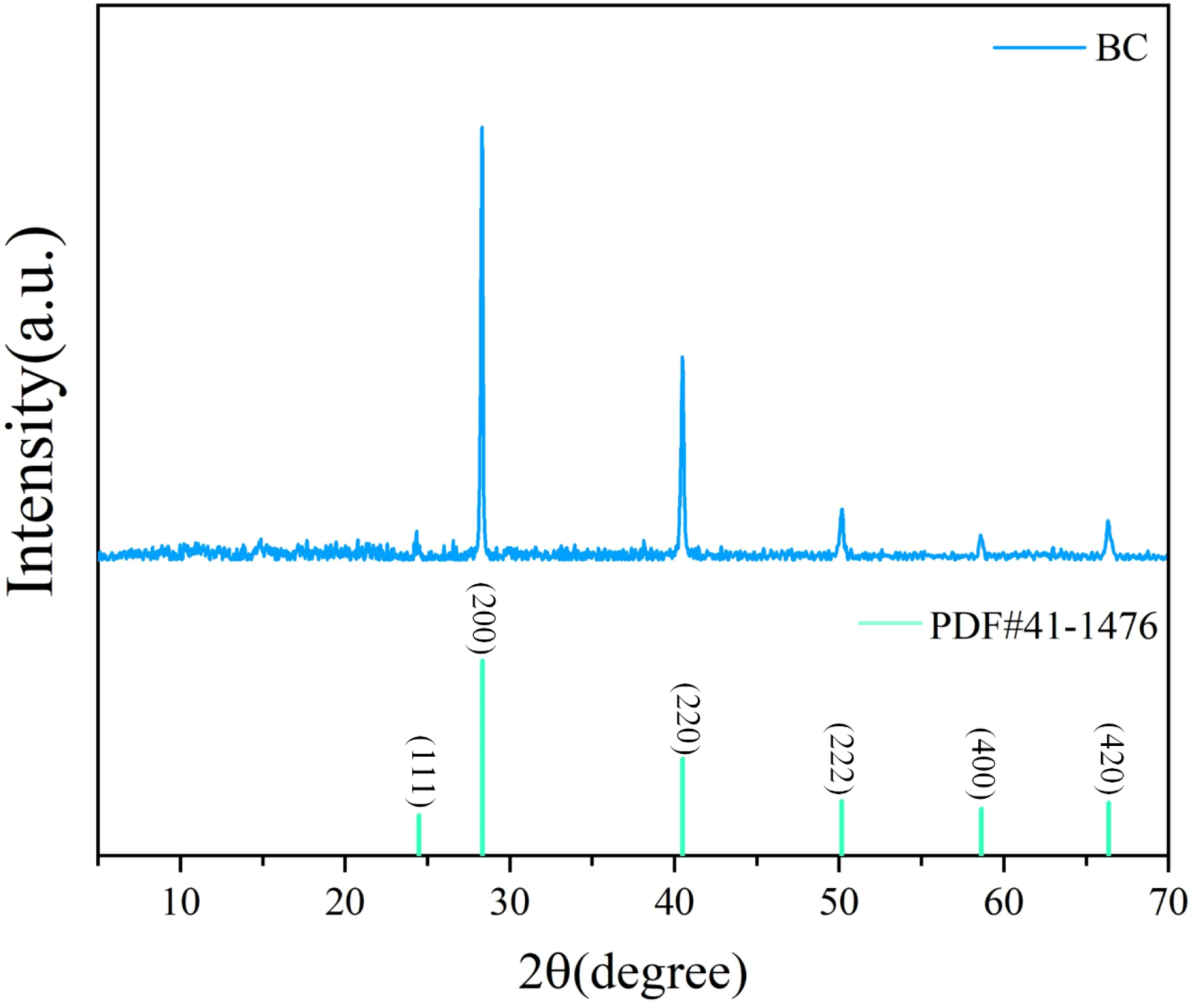
X-ray diffraction (XRD) pattern of *Wedelia trilobata*-derived biochar (WBC), illustrating the characteristic crystalline phases. Distinct peaks at 2θ values of 24.48°, 28.35°, 40.50°, 50.17°, 58.64°, and 66.38°correspond to the (111), (200), (220), (222), (400), and (420) planes, respectively, matching the standard pattern of potassium chloride (KCl, PDF#41-1476). The presence of KCl and other mineral phases indicates that the biochar retains plant-derived minerals after pyrolysis, which may contribute to nutrient enrichment and aid in chromium immobilization in hydroponic systems.

X-ray Diffraction (XRD) was employed to identify the crystalline phases present in the *Wedelia trilobata*-derived biochar sample, as shown in **Figure 3**. The XRD pattern exhibits several sharp and well-defined peaks, indicating a high degree of crystallinity in the material (Nanda et al., 2013) (Pasumarthi et al., 2024). Prominent diffraction peaks were observed at 2θ values of approximately 24.482°, 28.345°, 40.507°, 50.169°, 58.640°, and 66.381°, which correspond to the (111), (200), (220), (222), (400), and (420) crystal planes, respectively. These reflections closely match the standard pattern of potassium chloride (KCl), as referenced by the Powder Diffraction File PDF#41-1476. The presence of KCl may be attributed to the retention of native plant mineral components during pyrolysis, potentially enhancing the ionic exchange capacity and nutrient availability of the biochar when applied in hydroponic or soil systems.

**Figure 4:**
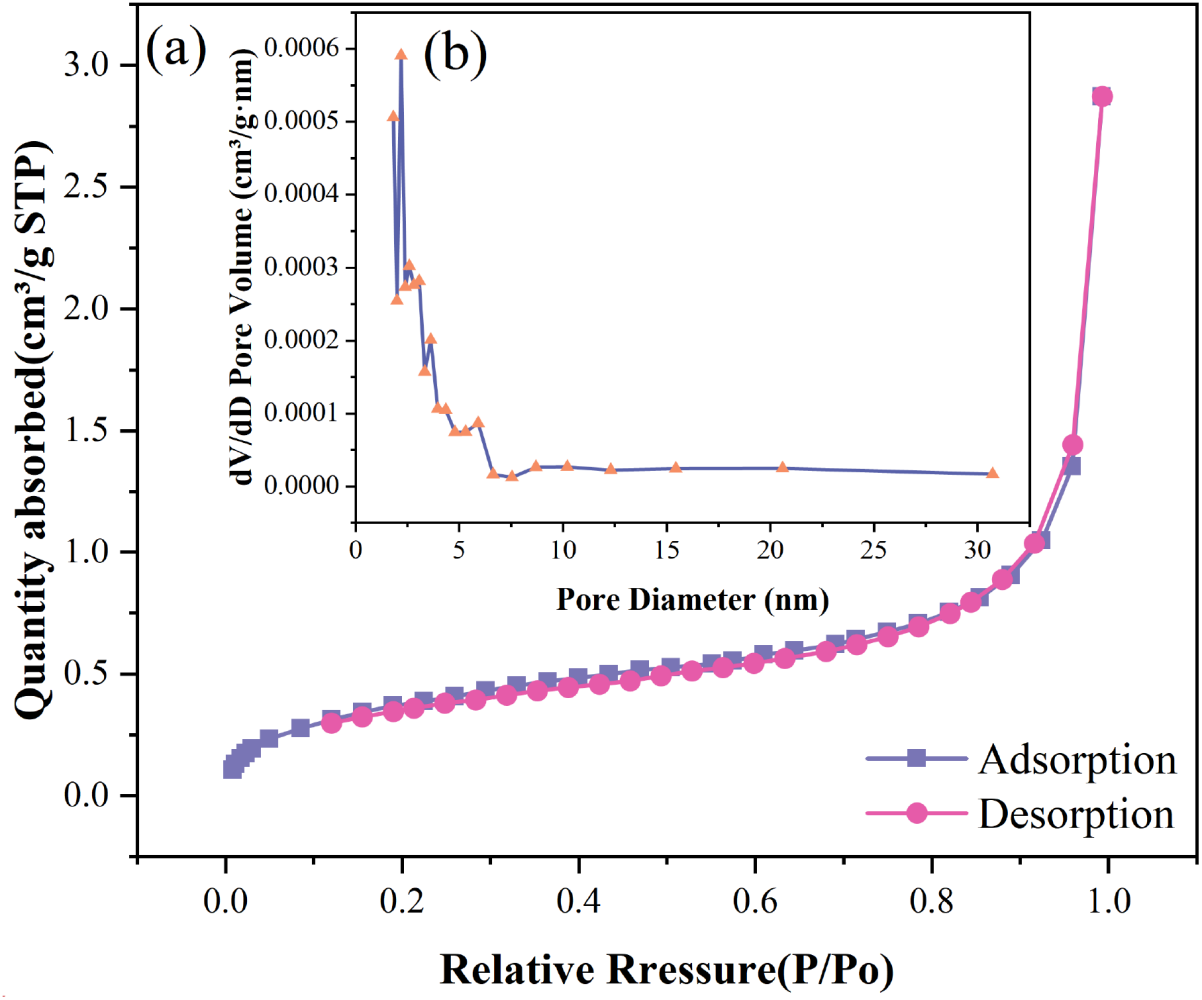
**(a)** Nitrogen adsorption–desorption isotherms of *Wedelia trilobata*-derived biochar (WBC), showing a type IV isotherm with an H3 hysteresis loop, indicative of mesoporous structure. **(b)** Barrett-Joyner-Halenda (BJH) pore size distribution curve, revealing a predominant pore diameter of ~2-5 nm.

The nitrogen adsorption-desorption isotherm and Barrett-Joyner-Halenda (BJH) pore size distribution curve of *Wedelia trilobata*-derived biochar (WBC) are presented in (**Figures 4a, b**), respectively. The isotherm exhibits a typical Type IV hysteresis loop, which is characteristic of mesoporous materials according to the IUPAC classification. This loop confirms the presence of mesopores and capillary condensation phenomena, indicating a porous structure well-suited for adsorption applications. The gradual increase in adsorption quantity with relative pressure (P/P_o_) and the sharp rise near P/P_o_ ≈ 1.0 suggest the presence of a combination of micro- and mesopores. The pore size distribution inset (**Figure 4b**) reveals that the majority of the pores fall below 10 nm, further supporting the mesoporous nature of WBC. The narrow distribution centered around ~3-5 nm indicates uniform pore development, which enhances surface accessibility for metal ions like Cr(VI). These findings are consistent with earlier studies highlighting the significance of surface area and mesoporosity in determining the sorption efficiency of biochar materials (Razali and Kamarulzaman, 2020, 2022; Wening and Herdyastuti, 2021). Thus, WBC’s structure-function relationship underscores its potential as a promising, sustainable amendment for heavy metal remediation and agricultural applications.

Potassium is one of the important nutrients for plant growth and metabolism (Kumar et al., 2020). Potassium is present in biochar in various forms, with water-soluble and non-water-soluble potassium being more common. Related studies have pointed out that preparation conditions such as pyrolysis temperature have a significant effect on the morphology and solubility of potassium present in biochar (Xiu et al., 2023). Biochar prepared at lower temperatures increases the proportion of water-soluble and exchangeable potassium (Bilias et al., 2023). It is widely believed that biochar contains large amounts of water-soluble potassium (Hossain et al., 2020). Water-soluble potassium dissolves rapidly in soil solution, providing a short-term source of easily absorbed potassium to meet the rapid needs of plants at specific growth stages. However, it is prone to migration in the soil solution and water loss to deeper layers of the soil or to the surrounding environment, resulting in reduced utilization (Cheng et al., 2023). In the study by (Kong et al., 2014) the water-soluble potassium content in biochar reached at least 47% of the total potassium content. The presence of insoluble potassium in biochar increases its slow-release capacity and reduces the leaching of potassium (Piash et al., 2021).

Insoluble potassium gradually releases potassium ions for plant uptake through slow dissolution or reaction with soil substances in the soil, so that the biochar can provide potassium to plants stably for a long time and reduce the problem of frequent fertilization due to rapid loss of potassium. From XRD analysis, the crystal structure of potassium-related minerals (e.g., KCl) in biochar is closely related to the solubility and release characteristics of potassium. The crystal structure integrity, the nature of crystal faces and the crystal grain size all affect the dissolution and release of potassium in biochar. When the crystal structure of potassium minerals in biochar is stable, potassium dissolution needs to overcome the higher energy barrier, showing a lower solubility and a slow-release rate.

### 3.2 Analysis of physiological and biochemical indicators

#### 3.2.1 Effect of biochar on chromium uptake

The chromium content in plants serves as an indicator of their ability to antagonize heavy metal stress, with lower chromium (Cr) accumulation reflecting greater resistance. Since the roots are the initial contact point for heavy metal ions, they play a critical role in Cr uptake (Saud et al., 2022). As shown in **Supplementary Figure S2**, under chromium (Cr) only treatment in group T1, the Cr content in the roots and shoots of cabbage was significantly higher. In line with our study, Bharani et al. (2015) noted that plant roots accumulate significantly more chromium than shoots, likely because roots are the primary site of contact and absorption. Similarly, Polti et al. (2011) reported that chromium mobility within roots is limited, leading to accumulation several times higher in roots than in other plant parts.

However, with the addition of *Wedelia trilobata* biochar in groups T2 (BC = 0.1 g/L), T3 (BC = 0.5 g/L), T4 (BC = 1 g/L), and T5 (BC = 3 g/L), a significant reduction in chromium content was observed. Specifically, Cr content in the roots decreased by 33.48%, 35.28%, 76.92%, and 97.12%, respectively, while in the shoots, it reduced by 33.35%, 35.10%, 77.19%, and 97.15%, respectively, compared to the T1 group. The incorporation of biochar significantly reduced Cr uptake in plant tissues, with the T5 treatment group demonstrating the most pronounced reduction of 97.15% in shoots and 97.12% in roots. The results of this study revealed a clear gradient in chromium removal with increasing biochar dosage, with the highest concentration treatment (T5) showing the most significant removal effect. This underscores the superior adsorption and immobilization capabilities of biochar for Cr. These findings align with the study by Bashir et al. (2021), which demonstrated that bagasse biochar effectively reduced Cr (VI and III) concentrations in maize roots and shoots, further supporting the results of this experiment. The removal of Cr(VI) by biochar increased with the increase of biochar concentration because the adsorption sites of Cr(VI) in solution increased with the increase of biochar concentration, which led to an increase in the total number of active sites, which resulted in easier adsorption of Cr(VI) and higher removal efficiency (Nasseh et al., 2017). The results of Yang et al. (2021) showed that the removal efficiency of Cr (VI) by corn stover biochar increased with increasing corn stover biochar concentration.

#### 3.2.2 Effect of biochar on pH and conductivity

As shown in **Supplementary Figure S3**, the pH and electrical conductivity (EC) of the hydroponic solution decreased by 1.04% and 4.68%, respectively, under chromium (Cr) only treatment in group T1. Chromium stress causes immediate acidification of nutrient solution (Arduini et al., 2006). In hydroponic systems, EC is often used to indicate the concentration of dissolved salts or ions in a solution; a low EC value may indicate nutrient deficiency or salt concentration due to water evaporation (Samarakoon et al., 2020). However, our study demonstrated that biochar amendments significantly raised the pH of the hydroponic solution, effectively neutralizing it and mitigating the toxic effects of chromium on cabbage. The addition of *Wedelia trilobata* biochar in groups T2, T3, T4, and T5 increased both pH and EC values as biochar concentration increased. The T5 group exhibited the most significant improvement, with pH and conductivity values rising by 35.97% and 16.79%, respectively. The increase in pH and EC values of water after biochar application is usually attributed to the accumulation of ash residues, which consist mainly of carbonates of alkali and alkaline earth metals, varying amounts of silica, heavy metals, sesquioxides, phosphates, and small amounts of organic matter and nitrogen (Raison, 1979). Another possible reason for the increase in soil pH is the high specific surface area and porosity of biochar, which increases the cation exchange capacity (CEC) in the reaction system (Nigussie et al., 2012). These findings align with a meta-analysis by Chen et al. (2018), which reported that biochar significantly increases soil pH, soil organic carbon (SOC), electrical conductivity (EC), and cation exchange capacity (CEC), enhancing the retention of heavy metals. The application of biochar significantly improved root zone chemistry by increasing both pH and electrical conductivity (EC), which enhanced nutrient availability and reduced Cr toxicity. The rise in pH is attributed to the alkaline nature of biochar containing basic cations (Jiang et al., 2012; Arwenyo et al., 2023), while the initial increase in EC reflects the release of soluble ions (Godlewska et al., 2021; Wang et al., 2024). These changes collectively promote better nutrient uptake and plant growth under stress conditions. This consistency further underscores the role of biochar in improving environmental conditions under heavy metal stress.

#### 3.2.3 Effect of biochar on photosynthetic efficiency of Chinese cabbage

**Figure 5:**
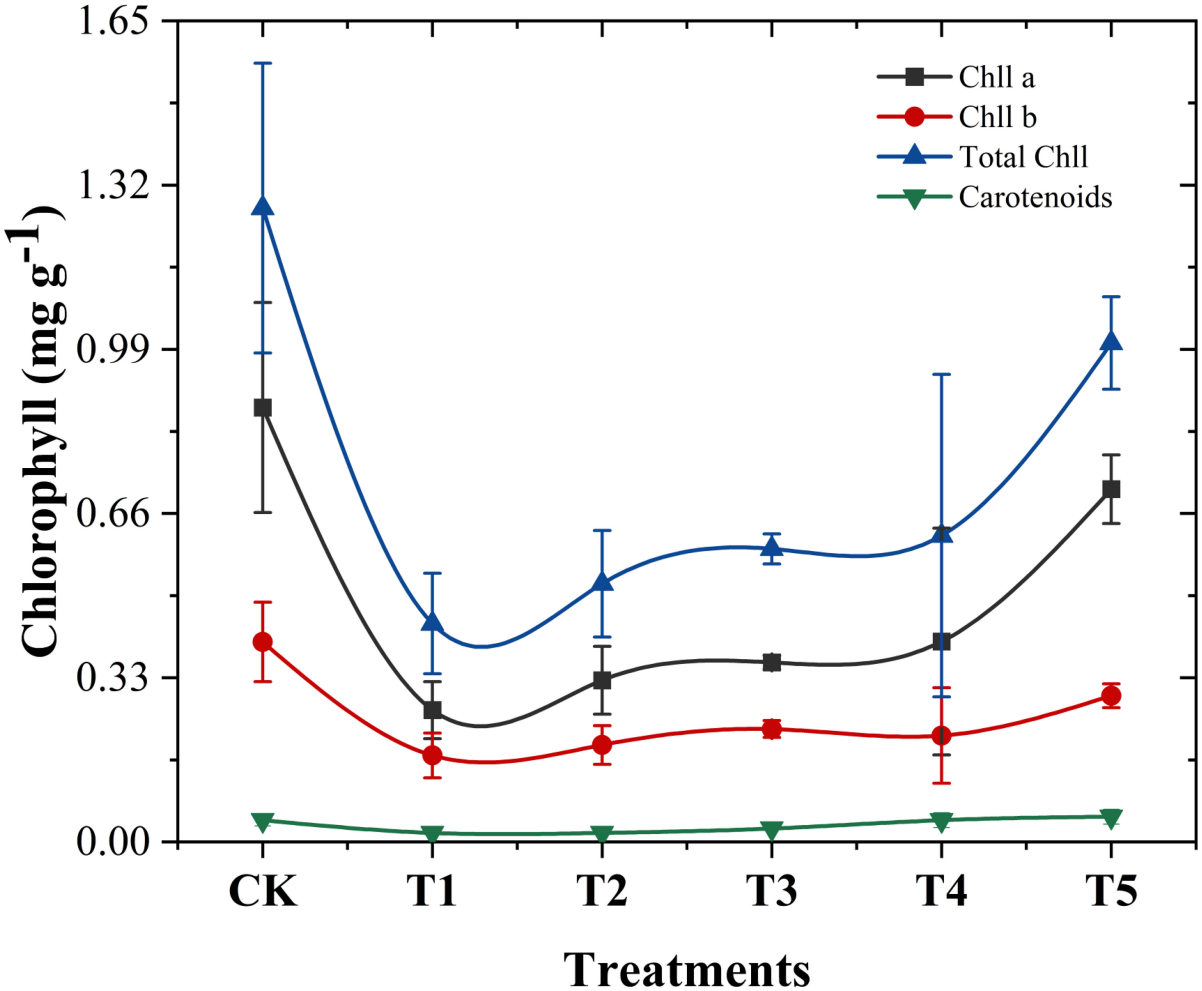
Impact of different *Wedelia trilobata*-derived biochar (WBC) concentrations on chlorophyll a, chlorophyll b, total chlorophyll and carotenoids content in the roots and shoots of Chinese cabbage (*Brassica rapa*) grown under chromium (Cr) stress in a hydroponic system. Plants were exposed to Cr (VI) (50 mg L^-1^) with or without WBC at varying concentrations (0, 0.1, 0.5, 1.0, and 3.0 g L^-1^) for 7 days. Error bars indicate standard deviation (SD) of the mean (n = 3). Results show that higher WBC application significantly restored chlorophyll levels compared to Cr-only treatment, particularly in shoots.

Chlorophylls and carotenoids, located in plant chloroplasts, play a critical role in the absorption and transfer of light energy during photosynthesis (Zulfiqar et al., 2021; Simkin et al., 2022). As shown in **Figure 5**, chromium stress in the T1 treatment significantly reduced chlorophyll A, chlorophyll B, total chlorophyll and carotene contents in cabbage leaves by 69.64%, 56.72%, 65.54%, and 59.09%, respectively, compared to the control (no Cr and biochar amendments). In line with our study Sallah-Ud-Din et al. (2017) reported decreased pigment content under increased chromium stress. This reduction may result from chromium ions disrupting plant cell membrane structures, increasing cell permeability, and causing the leakage of substances, including pigments like chlorophyll and carotenoids (Dai and Shan, 2019). Chromium’s negative impact on photosynthesis, including disruptions in electron transfer, CO_2_ fixation, enzyme activity, and photophosphorylation, is well documented (Zaheer et al., 2022).

However, the addition of *Wedelia trilobata* biochar in T2 to T5 treatments reversed this trend, promoting an increase in photosynthetic pigment content. Specifically, T2 treatment increased chlorophyll A, chlorophyll B, and total chlorophyll content by 22.64%, 12.07%, and 18.22%, respectively, while carotene content remained unchanged. Carotenoids act as accessory pigments, aiding in light harvesting and protecting the photosynthetic apparatus from damage caused by high-intensity light, thereby enhancing the efficiency of photosynthesis (Hashimoto et al., 2016). However, in our present study, in our present study, similar to the results of Islam, constant carotenoid content under chromium stress may support the avoidance strategy against chromium toxicity (Islam et al., 2022). T3 treatment resulted in increases of chlorophyll a by 36.23%, chlorophyll b by 30.46%, total chlorophyll by 34.17%, and carotene by 50% compared to metal only treatment (T1). T4 treatment demonstrated further improvement, with increases of 52.08%, 22.99%, 40.32%, and 144.44%, respectively. The most significant enhancement was observed in the T5 treatment, where chlorophyll A, chlorophyll B, total chlorophyll, and carotene content increased by 167.55%, 68.97%, 128.47%, and 183.33%, respectively, compared to plants treated with Cr only. It is clear that high concentrations of biochar significantly increase chlorophyll content, especially under T5 treatment. This improvement is likely due to biochar’s ability to adsorb chromium, reducing its availability and toxicity to plants (Lucchini et al., 2014; Wang et al., 2015). Similar findings He et al. (2020) revealed that biochar additives improved photosynthetic rates, stomatal conductance, transpiration rates, water use efficiency, and chlorophyll concentrations, aligning with the results of this study.

#### 3.2.4 Effect of biochar on plant growth indicators

**Figure 6:**
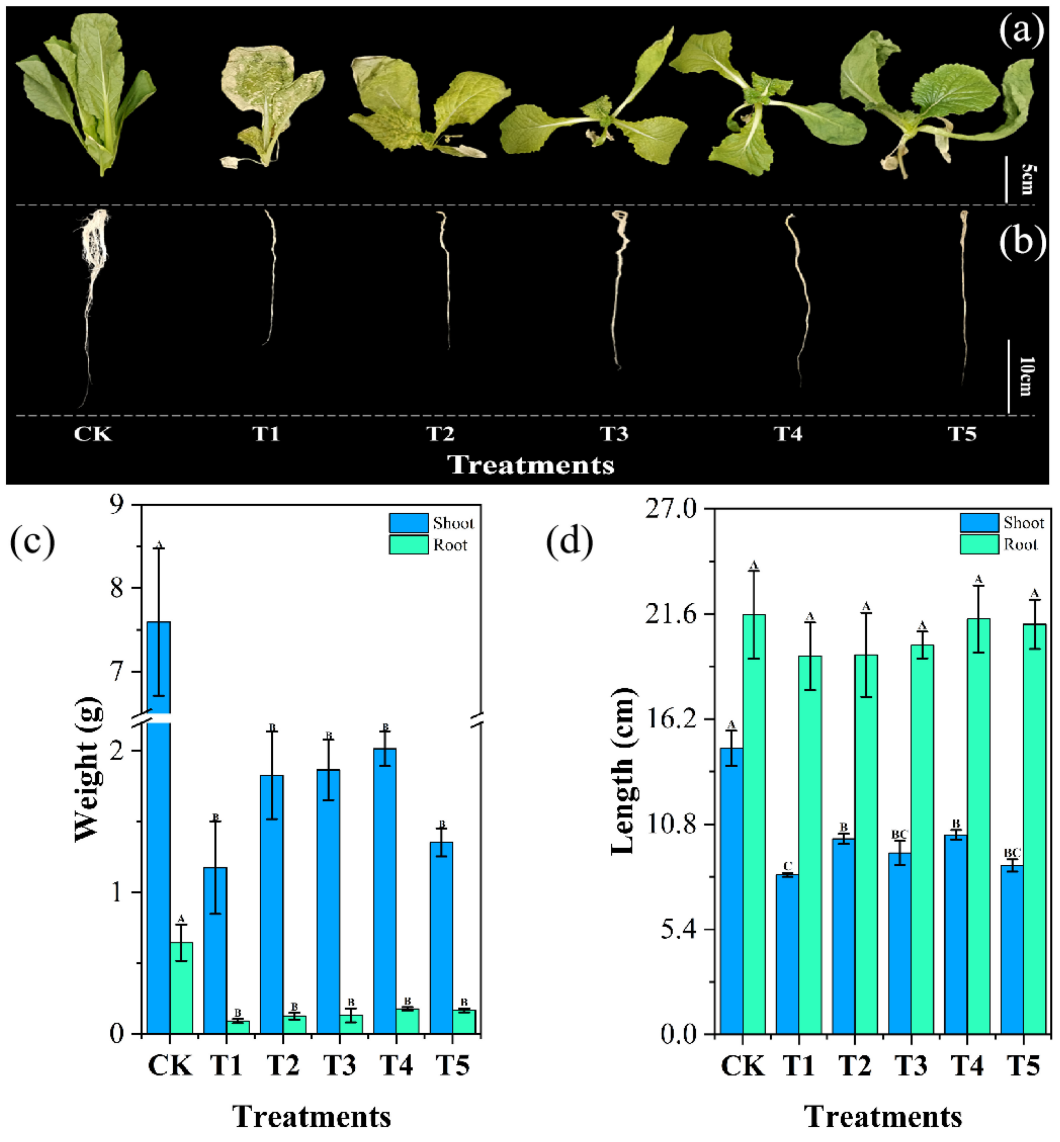
Impact of different *Wedelia trilobata*-derived biochar (WBC) concentrations on the morphological characteristics of Chinese cabbage (*Brassica rapa*) grown under chromium (Cr) stress in a hydroponic system. **(a)** shoot development, **(b)** root development, **(c)** fresh weight of shoots and roots, and **(d)** total plant length. Plants were treated with Cr (VI) alone (50 mg L^-1^) or Cr (VI) plus WBC at varying concentrations (0, 0.1, 0.5, 1, and 3 g L^-1^) for 7 days. Error bars represent standard deviation (SD) of the mean (n = 3), and different capital letters indicate statistically significant differences among treatments based on Tukey’s HSD test (p < 0.05). WBC application mitigated Cr-induced growth inhibition, with higher concentrations promoting greater biomass accumulation and length.

Chromium exerts a strong inhibitory effect on cabbage growth, significantly reducing biomass accumulation (Li et al., 2019). In our study, as shown in **Figure 6**, root and shoot weights were considerably higher in the CK group than in the five treatment groups. Specifically, in the T1 group, chromium exposure led to a significant reduction in root and shoot weights by 85.71% and 84.53%, respectively, compared to CK. However, biochar amendment enhanced biomass accumulation, increasing root and shoot weights by 36.96% and 55.49% in T2, 42.39% and 58.89% in T3, 92.39% and 71.57% in T4, and 80.43% and 15.32% in T5, respectively, relative to T1.

Similarly, root length in T1 decreased by 9.85% compared to CK, though this reduction was not statistically significant, whereas shoot length was significantly reduced by 44.16%. With biochar addition, root length in T2, T3, T4, and T5 increased by 0.4%, 2.98%, 9.82%, and 8.46%, respectively, relative to T1, though these increases were not statistically significant. However, shoot length significantly improved by 22.49% in T2, 13.68% in T3, 24.93% in T4, and 5.83% in T5. The suppression of root growth under chromium stress is mainly attributed to its interference with root cell division, elongation, and the cell cycle (Nagarajan and Ganesh, 2014). At the cellular level, chromium infiltrates root cells, disrupting intracellular signaling pathways and impairing the expression of genes essential for cell division and elongation. This disruption hampers normal cellular processes, leading to stunted root growth. Additionally, exposure to chromium-containing solutions can cause tissue collapse in plant roots, impairing their ability to absorb water and nutrients. These physiological disturbances ultimately reduce biomass accumulation and other growth indicators (Danish et al., 2019).

In this study, we observed that excessive chromium in the nutrient solution caused significant shoot damage in Chinese cabbage, which was more pronounced than root damage. This effect may be partly due to chromium accumulation in leaves, leading to impaired photosynthesis, oxidative stress, and chloroplast damage (Gill et al., 2016). Our data indicate that cabbage leaves, rather than roots, are highly sensitive to chromium toxicity. Cabbage plants tend to limit Cr accumulation in aerial parts by sequestering it in the roots, where it is less toxic (Perez-Millan et al., 2021). Consequently, the addition of *Wedelia trilobata* biochar had a more pronounced effect on shoot growth.

#### 3.2.5 Effect of biochar on plant nutrient content

Nitrogen (N), phosphorus (P), potassium (K), calcium (Ca), and magnesium (Mg) are essential nutrients that play a crucial role in plant growth, metabolism, yield, and resistance to biotic and abiotic stresses (Sung et al., 2018; de Bang et al., 2021). **Supplementary Figure S4** illustrates the variations in nutrient content in cabbage shoots across six different treatment groups. Under chromium stress in the T1 group, the contents of N, P, K, Ca, and Mg decreased significantly by 14.90%, 59.65%, 24.55%, 80.10%, and 34.96%, respectively, compared to the CK group. This decline underscores the detrimental impact of chromium on nutrient accumulation in cabbage. Chromium stress reduces nutrient availability and uptake due to its ionic similarity to elements such as calcium, potassium, and magnesium, leading to competitive inhibition during nutrient absorption (Oliveira, 2012; Kapoor et al., 2022). Studies have shown that plants exposed to hexavalent chromium experience reduced uptake of key macronutrients, including nitrogen, phosphorus, and potassium (Srivastava et al., 2021). Chromium competes for adsorption sites, limiting nutrient transport and absorption in plants (Handa et al., 2019; Kapoor et al., 2022).

However, compared to T1, treatments T2, T3, and T4 exhibited significant increases in nitrogen content by 6.52%, 14.26%, and 20.79%, respectively, while T5 showed a non-significant increase of 0.59%. Significant improvements in phosphorus content were also observed in T2, T3, T4, and T5. Potassium levels increased significantly by 7.01%, 18.25%, 27.22%, and 10.19% in T2, T3, T4, and T5, respectively. Notably, calcium content increased by 353.24%, 473.52%, 478.99%, and 355.49%, while magnesium content showed significant enhancements of 40.08%, 59.81%, 97.86%, and 79.24% in the same treatments. Overall, biochar-treated groups (T2-T5) exhibited an upward trend in nutrient content, with T4 demonstrating the most substantial improvement. These finding highlights biochar’s potential to counteract nutrient depletion caused by chromium stress. Biochar has been shown to reduce heavy metal bioavailability by adsorbing metal ions, thereby mitigating their toxic effects on plants (Ahmad et al., 2014). In this study, biochar alleviated chromium-induced stress by adsorbing chromium ions, thereby enhancing the plant’s ability to absorb essential nutrients. Additionally, biochar itself serves as a source of macronutrients such as potassium, calcium, and phosphorus, which may directly contribute to improved nutrient content in plants (Limwikran et al., 2019; Chhimwal et al., 2022). Its ability to supply both macronutrients and micronutrients further explains the observed enhancement in nutrient accumulation in cabbage shoots (Choudhary et al., 2021).

#### 3.2.6 Effect of biochar on the redox of cabbage under chromium stress

**Figure 7:**
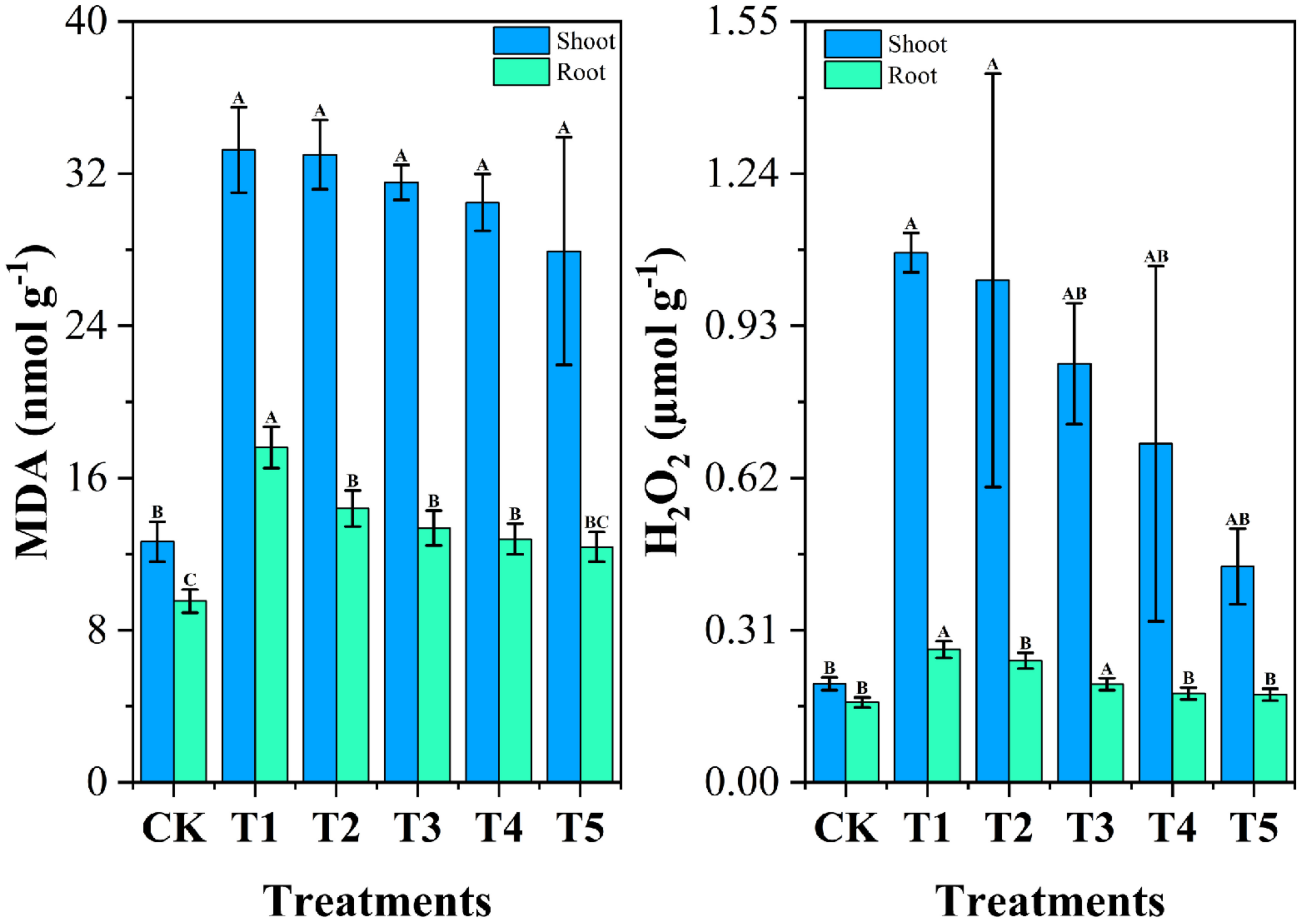
Impact of different *Wedelia trilobata*-derived biochar (WBC) concentrations on oxidative stress markers-malondialdehyde (MDA) and hydrogen peroxide (H_2_O_2_)-in roots and shoots of Chinese cabbage (*Brassica rapa*) grown under chromium (Cr) stress in a hydroponic system. Plants were exposed to Cr (VI) alone (50 mg L^-1^) or Cr (VI) plus WBC at concentrations of 0, 0.1, 0.5, 1, and 3 g L^-1^ for 7 days. MDA content indicates the extent of lipid peroxidation, while H_2_O_2_ levels reflect oxidative stress. Error bars represent standard deviation (SD) of the mean (n = 3). Different capital letters indicate statistically significant differences among treatments according to Tukey’s HSD test (p < 0.05). WBC application reduced both MDA and H_2_O_2_ levels, indicating mitigation of Cr-induced oxidative damage.

Malondialdehyde (MDA) and hydrogen peroxide (H_2_O_2_) are widely recognized as biomarkers of oxidative stress in plants (Zafar et al., 2024). **Figure 7** illustrates the effects of chromium stress treatment on oxidative stress indicators, malondialdehyde (MDA), and hydrogen peroxide (H_2_O_2_), in the roots and shoots of cabbage plants. Under chromium stress (T1 group), MDA content significantly increased by 84.64% in roots and 162.57% in shoots compared to the CK group. Similarly, the content of H_2_O_2_ in T1 was significantly elevated by 66.26% in roots and 436.82% in shoots compared to CK. The elevated levels of MDA and H_2_O_2_ under chromium stress in this study reflect the oxidative damage caused by reactive oxygen species (ROS) production and lipid peroxidation. Chromium toxicity disrupts cellular metabolism, induces oxidative stress, and damages membrane-bound organelles, ultimately impairing plant growth and biomass accumulation (Gautam et al., 2020; Hassan et al., 2022). Increased MDA and H_2_O_2_ levels under chromium stress have been previously observed in various plants, including cauliflower, due to heightened ROS production and compromised oxidative defense mechanisms (Ahmad et al., 2020). However, biochar treatments (T2-T5) showed a gradual decrease in MDA levels, with T5 demonstrating the most effective reduction of 29.66% in roots and 15.98% in shoots.

In the biochar-treated groups, H_2_O_2_ levels decreased progressively with increasing biochar concentration, with T5 showing the most pronounced reductions of 33.95% in roots and 59.22% in shoots compared to T1. This is because the addition of biochar likely alleviates chromium-induced stress by adsorbing heavy metal ions, thereby reducing ROS production and protecting cell membrane integrity through the inhibition of lipid peroxidation (Fu et al., 2023). Previous studies have shown that biochar applications can lower MDA and H_2_O_2_ levels in plants under heavy metal stress, such as cadmium in wheat and various heavy metals in Chinese mustard (Awad et al., 2022; Hussain et al., 2022). This study corroborates these findings, demonstrating the potential of biochar in alleviating oxidative damage and improving plant resilience under heavy metal stress.

#### 3.2.7 Effect of biochar on the activities of antioxidant enzymes (SOD, CAT, POD) in Chinese cabbage

**Figure 8:**
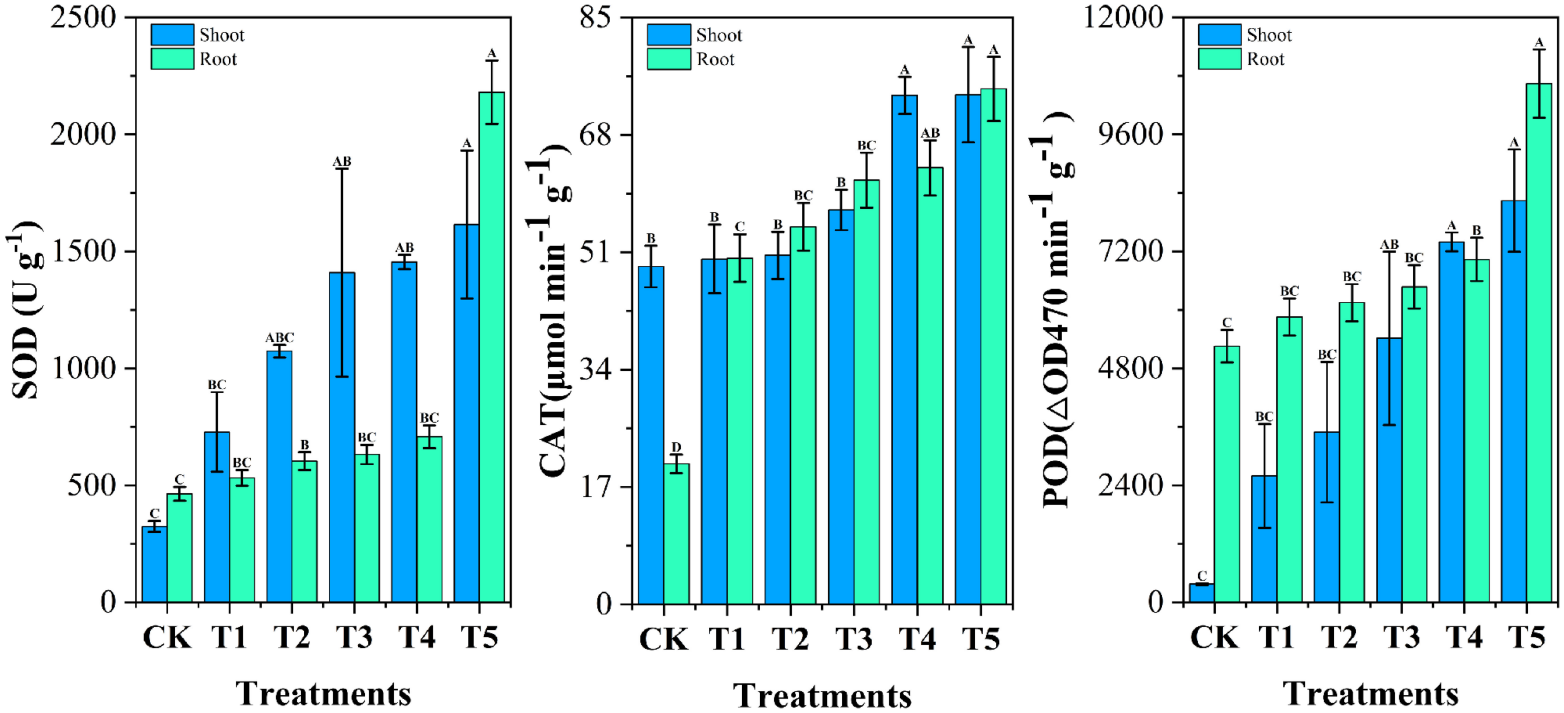
Impact of different *Wedelia trilobata*-derived biochar (WBC) concentrations on antioxidant enzyme activities-superoxide dismutase (SOD), catalase (CAT), and peroxidase (POD)-in roots and shoots of Chinese cabbage (*Brassica rapa*) grown under chromium (Cr) stress in a hydroponic system. Treatments included Cr (VI) alone (50 mg L^-1^) and Cr (VI) plus WBC at concentrations of 0, 0.1, 0.5, 1, and 3 g L^-1^ for 7 days. Increased SOD, CAT, and POD activities indicate enhanced reactive oxygen species (ROS) scavenging capacity. Error bars represent standard deviation (SD) of the mean (n = 3). Different capital letters denote statistically significant differences among treatments according to Tukey’s HSD test (p < 0.05).

As shown in **Figure 8**, Cr affected the activities of antioxidant enzymes such as SOD, CAT and POD in roots and shoots of Chinese cabbage. Chromium stress enhanced the activities of various antioxidant enzymes in cabbage in roots and shoots as compared to control, SOD, CAT and POD activities were increased (14.73% and 124.44%, 146.66% and 2.23%, 11.42% and 594.71%, respectively). It is well known that biotic and abiotic stresses disrupt redox homeostasis in plant cells and induce the accumulation of reactive oxygen species (ROS) (Ali et al., 2011). Plant cells and their organelles, such as chloroplasts, mitochondria, and peroxisomes, employ an antioxidant defense system to protect themselves from ROS-induced oxidative stress (Gill and Tuteja, 2010). The results showed that the activities of SOD, CAT and POD in plants increased significantly under chromium stress, which is the change of antioxidant defense system in response to external chromium stress (Anjum et al., 2017). WBC treatment increased the activities of antioxidant enzymes SOD, POD and CAT in cabbage under chromium stress conditions. The elevated activities of antioxidant enzymes indicated that WBC treatment could improve plant tolerance to chromium and maintain the plant’s own redox balance by inhibiting the excess ROS produced by the plant under chromium stress. The results of related studies showed that the use of rice straw biochar improved the performance of antioxidant enzymes such as SOD, POD and CAT and successfully ameliorated the adverse effects of Cd on wheat, which is consistent with the results of the present study (Hussain et al., 2022).

#### 3.2.8 Effect of biochar on proline activity of Chinese cabbage

Proline is considered to be a multifunctional molecule that accumulates in high concentrations under various abiotic stresses (Kavi Kishor and Sreenivasulu, 2014). Proline acts as an osmoprotector, maintaining cell hydration and expansion, and as an antioxidant, clearing reactive oxygen species (ROS) to protect cell components (Tiwari, 2024). It serves multiple functions, including osmoregulation, enzyme protection, detoxification of harmful reactive oxygen species (ROS), and maintenance of protein synthesis (Bashir et al., 2021). As shown in **Supplementary Figure S5**, proline content in the roots and shoots of plants under chromium stress (T1) increased markedly by 131.07% and 3406.74%, respectively, compared to CK. This substantial accumulation likely reflects the plant’s defense mechanism, as many plants synthesize compatible osmotic solutes like proline to ensure membrane stability and osmoregulatory functions during stress (Sharma et al., 2019). Proline appears to alleviate metal toxicity by acting as a metal chelator and stabilizing proteins (Mansoor et al., 2021). Furthermore, proline accumulation has been observed to counter various biotic and abiotic stresses, including salinity, drought, temperature extremes, heavy metals, and pathogen attacks (Hayat et al., 2012). The findings of Hemadri Reddy et al. (2024) who reported increased proline levels in mung bean seedlings exposed to cadmium, align with the results of this study, indicating a consistent role of proline under heavy metal stress.

However, in the roots and shoots of cabbage tissues treated with biochar, proline content decreased significantly across all treatments compared to T1. Specifically, proline content in T2 was reduced by 58.91% and 18.91%; in T3 by 58.36% and 77.91%; in T4 by 59.44% and 78.60%; and in T5 by 62.85% and 79.78%, respectively. This reduction suggests a decreased stress response in the plants due to the alleviating effects of biochar treatment. Similar findings were reported by Kumar et al. (2019) who observed reduced proline levels in maize with biochar application. Awad et al. (2022) also found that 5% rice husk biochar reduced proline content in Chinese mustard plants under heavy metal stress. Similarly, Mokarram-Kashtiban et al. (2019) reported that biochar application to hornbeam (*Carpinus betulus* L.) reduced proline content in white willow seedlings subjected to heavy metal stress (Cu, Pb, and Cd). These findings align with the results of this study, suggesting that biochar improves photosynthesis in plants, enhancing their energy and resources for maintaining normal physiological functions. This allows plants to rely less on proline accumulation as a defense mechanism against chromium stress.

#### 3.2.9 Effect of biochar on nutritional quality (soluble sugar, soluble protein) of cabbage

In the roots and shoots of cabbage plants under chromium stress, soluble protein content decreased by 47.39% and 15.47%, respectively, in T1 compared to CK (**Supplementary Figure S6**). However, with biochar treatments, soluble protein content increased gradually compared to T1: by 4.74% and 0.39% in T2, 17.45% and 2.31% in T3, 8.96% and 22.04% in T4, and 17.43% and 28.13% in T5. Despite these increases, none reached a significant level. Similarly, soluble sugar content in the roots and shoots decreased by 60.35% and 47.82%, respectively, in T1 compared to CK. Under biochar treatments, soluble sugar content increased progressively: by 11.20% and 152.22% in T2, 24.32% and 229.64% in T3, 35.23% and 284.10% in T4, and 78.09% and 502.35% in T5, compared to T1.

The reduction in soluble protein and sugar content under chromium stress indicates that chromium adversely affects protein synthesis and sugar metabolism, depleting energy reserves and diminishing stress response capabilities in plants. Chromium stress likely disrupts cellular metabolism and inhibits protein synthesis, causing a significant decline in soluble protein levels, possibly due to denaturation induced by the heavy metal (Xie et al., 2019). Simultaneously, soluble sugars, as vital energy sources, are utilized preferentially to maintain osmotic balance, provide energy, and support stress responses, accelerating their consumption (Du et al., 2020). Biochar treatments from T2 to T5 resulted in gradual increases in soluble protein and sugar content, suggesting an alleviation of chromium stress. This may involve the production of organic acids, glutathione, phytochelatins, metallothioneins, heat shock proteins, proline, and phytohormones, reducing the plant’s stress perception and response at cellular and whole-plant levels (Thakur et al., 2022). Additionally, biochar could optimize resource allocation, enabling plants to prioritize essential life activities and protein metabolism, leading to an increase in soluble protein levels, albeit insufficient to reach significant thresholds, indicating a conserved plant response to heavy metal stress. The increase in soluble sugar content with biochar addition could be due to enhanced photosynthesis under improved growth conditions, promoting sugar synthesis. Adjustments in metabolic pathways may also facilitate the storage of photosynthetic products as soluble sugars. Hussain et al. (2022) reported increased soluble sugar content in cadmium-stressed plants with biochar application. Similarly, Sami et al. (2023) observed that induced Cr stress reduced soluble proteins and sugars in spinach, but the addition of cotton stalk biochar increased both, aligning with the findings of this study.

### 3.3 Correlation matrix analysis

**Figure 9:**
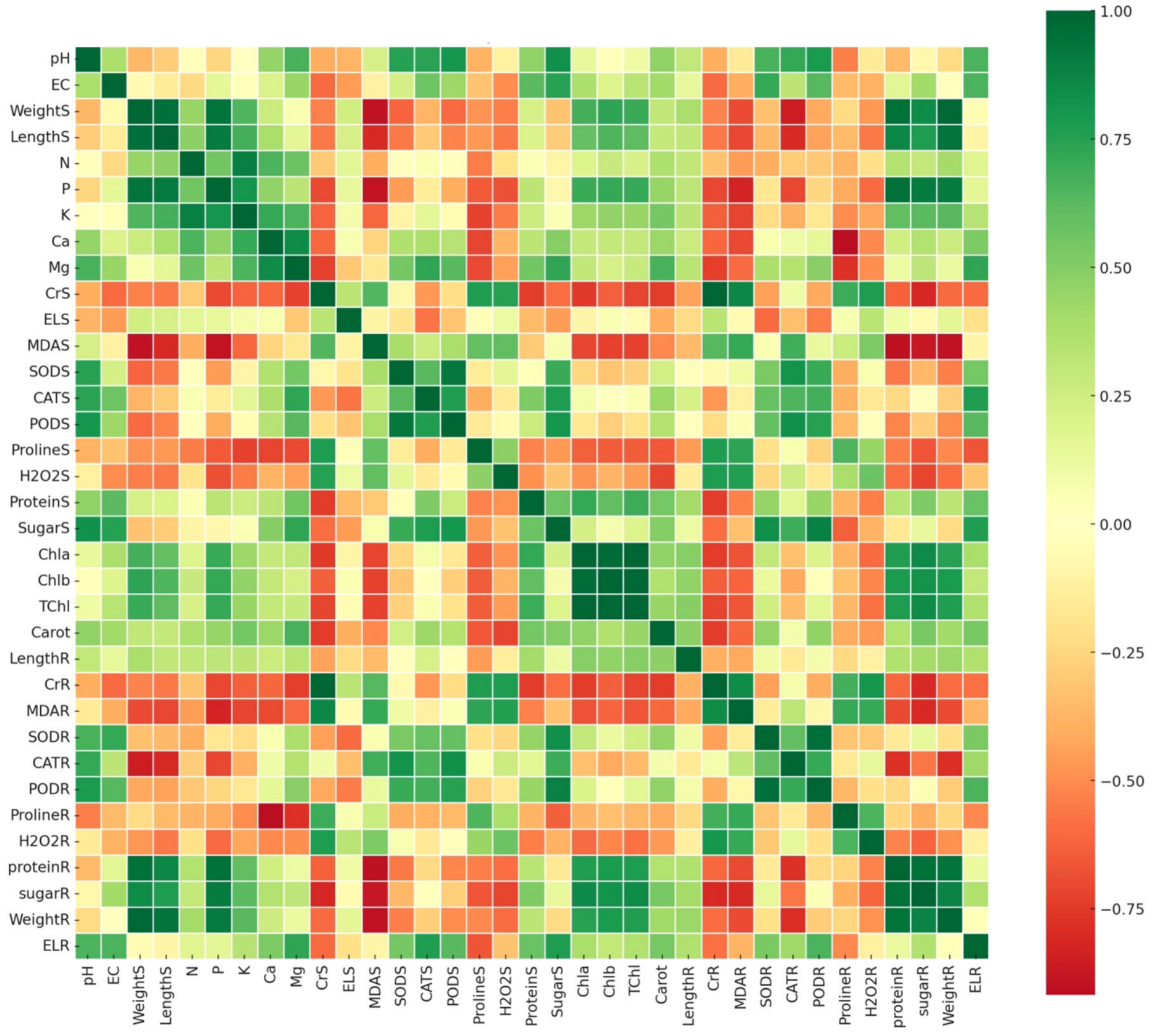
Pearson correlation heatmap of physiological, biochemical, and growth-related traits in Chinese cabbage (*Brassica rapa*) grown under chromium (Cr) stress with and without *Wedelia trilobata*-derived biochar (WBC) treatments in a hydroponic system. Traits include nutrient content (Ca, Mg, etc.), oxidative stress markers (malondialdehyde (MDA), hydrogen peroxide (H_2_O_2_)), antioxidant enzyme activities (superoxide dismutase (SOD), catalase (CAT), peroxidase (POD)), and growth parameters (shoot/root length, biomass). Green shades indicate strong positive correlations (r → +1), while red shades represent strong negative correlations (r → –1).

The correlation matrix analysis highlighted strong interrelationships among physiological, biochemical, and growth traits in *Brassica rapa* under chromium (Cr) stress and biochar treatments, revealing that shoot weight (WeightS) was positively correlated with shoot length, macronutrient levels (N, P, K), and photosynthetic pigments (TChl, Chla, Chlb) (**Figure 9**), suggesting that improved nutrient availability and photosynthetic efficiency contributed significantly to biomass accumulation consistent with previous findings on biochar-enhanced nutrient uptake under heavy metal stress (Verlinden et al., 2018; Chtouki et al., 2022). Antioxidant enzymes (SODR, CATR, PODR) were positively intercorrelated, indicating coordinated oxidative defense, though their weak or negative correlation with shoot biomass suggests they act as stress indicators rather than growth promoters, while oxidative markers like malondialdehyde (MDAS) and hydrogen peroxide (H_2_O_2_) were negatively associated with growth traits, confirming their role in stress-related damage. Additionally, osmolytes such as proline and soluble sugars showed moderate positive correlations with antioxidants and pigments, hinting at their dual role in ROS detoxification and photoprotection under Cr stress. Secondary nutrients like calcium and magnesium also correlated positively with shoot biomass and chlorophyll content, reinforcing their contribution to structural and metabolic resilience (Jin et al., 2023), while phosphorus emerged as a key driver of growth recovery under Cr exposure. Collectively, these correlations support the conclusion that *Wedelia trilobata*-derived biochar enhances nutrient assimilation and modulates stress-responsive metabolic pathways, reducing oxidative damage and promoting growth, thereby positioning it as an effective and sustainable amendment for Cr-contaminated hydroponic systems.

### 3.4 Principal component analysis

Principal component analysis (PCA) was conducted to evaluate the multivariate variation among treatments, with PC1 and PC2 accounting for 43.07% and 28.67% of the total variance, respectively (cumulative 71.74%). The PCA biplot revealed clear separation between treatments, with distinct clustering patterns and labeled centroids indicating that higher WBC concentrations (T4 and T5) were associated with more favorable physiological and biochemical traits compared to the chromium-only treatment (T1). The Cr-only treatment (T2) clustered far from the control (T1) (**Supplementary Figure S7**), reflecting the detrimental effects of Cr toxicity on plant health, such as reduced biomass, impaired nutrient uptake, and elevated oxidative stress. In contrast, treatments with *Wedelia trilobata*-derived biochar (T3-T5) progressively shifted toward the control cluster, suggesting a dose-dependent alleviation of Cr-induced stress through improvements in antioxidant enzyme activity, photosynthetic pigment content, and nutrient assimilation. These results support the role of biochar in mitigating abiotic stress by modulating plant metabolic pathways and improving soil or hydroponic nutrient dynamics, consistent with previous findings (Barzana et al., 2021). The PCA thus confirms biochar’s potential as a sustainable amendment to restore plant functionality under heavy metal stress.

## 4 Conclusions

This study confirms that *Wedelia trilobata*-derived biochar (WBC) is a promising strategy for alleviating chromium (Cr)-induced abiotic stress in hydroponically grown Chinese cabbage (*Brassica rapa*). WBC demonstrated a strong Cr immobilization capacity, reducing Cr accumulation in shoots and roots by over 97% at a 3 g/L application rate. It significantly improved plant growth and physiological performance, including enhanced root and shoot biomass, increased chlorophyll and carotenoid content, and strengthened antioxidant defense systems. WBC also reduced oxidative stress markers and promoted nutrient uptake, with substantial increases in calcium and magnesium concentrations. The findings contribute valuable insights into biochar-based abiotic stress alleviation strategies, supporting its broader application in sustainable agriculture and environmental remediation. Future studies should evaluate its performance in soil-based systems and explore its interactions with soil microbiota to enhance its practical utility. Future studies should explore the synergistic effects of combining WBC with microbial inoculants to enhance remediation efficiency and plant health. Additionally, field-scale trials are recommended to validate the practical applicability of WBC under natural environmental conditions and in various crop production systems.

## Data Availability

The datasets presented in this study can be found in online repositories. The names of the repository/repositories and accession number(s) can be found in the article/**Supplementary Material**.
