# Supplementary material for: Wedelia trilobata-derived biochar mitigates chromium toxicity and improves physiological performance in hydroponically grown Chinese cabbage
Source: Front Plant Sci. 2025 Sep 9;16:1624352. doi: 10.3389/fpls.2025.1624352 (PMC12454059; doi:10.3389/fpls.2025.1624352)
Supplement: Supplementary file 1 [file DataSheet1.docx]

***Supplementary information***

***Wedelia trilobata*-derived biochar mitigates chromium toxicity and improves physiological performance in hydroponically grown Chinese cabbage**

Qin Fengyue^1,2#^, Weidong Li^1,2#^, Menglu Dong^1,2^, Shuangqi Yue^1,2^, Guojie Weng^1,2^, Mingxuan Wang^1,2^, Xinyu Shan^1,2^, Waqas Ahmed^1,2^, Jiechang Weng^3^*, Sajid Mehmood^1,2^*.

^1^Center for Eco-Environment Restoration of Hainan Province, School of Ecology, Hainan University, Haikou, 570228, China

^2^School of Topical Agriculture and Foresty, Hainan University, Haikou, 570228, China

^3^Hainan Provincial Ecological and Environmental Monitoring Center, Haikou, 570228, China.

**#These authors contributed equally to this paper**

***Corresponding author:**

**Jiechang Weng;** E-mail: [wjchang9033@126.com,](mailto:wjchang9033@126.com,)

**Sajid Mehmood;** E-mail: [drsajid@hainanu.edu.cn](mailto:drsajid@hainanu.edu.cn)


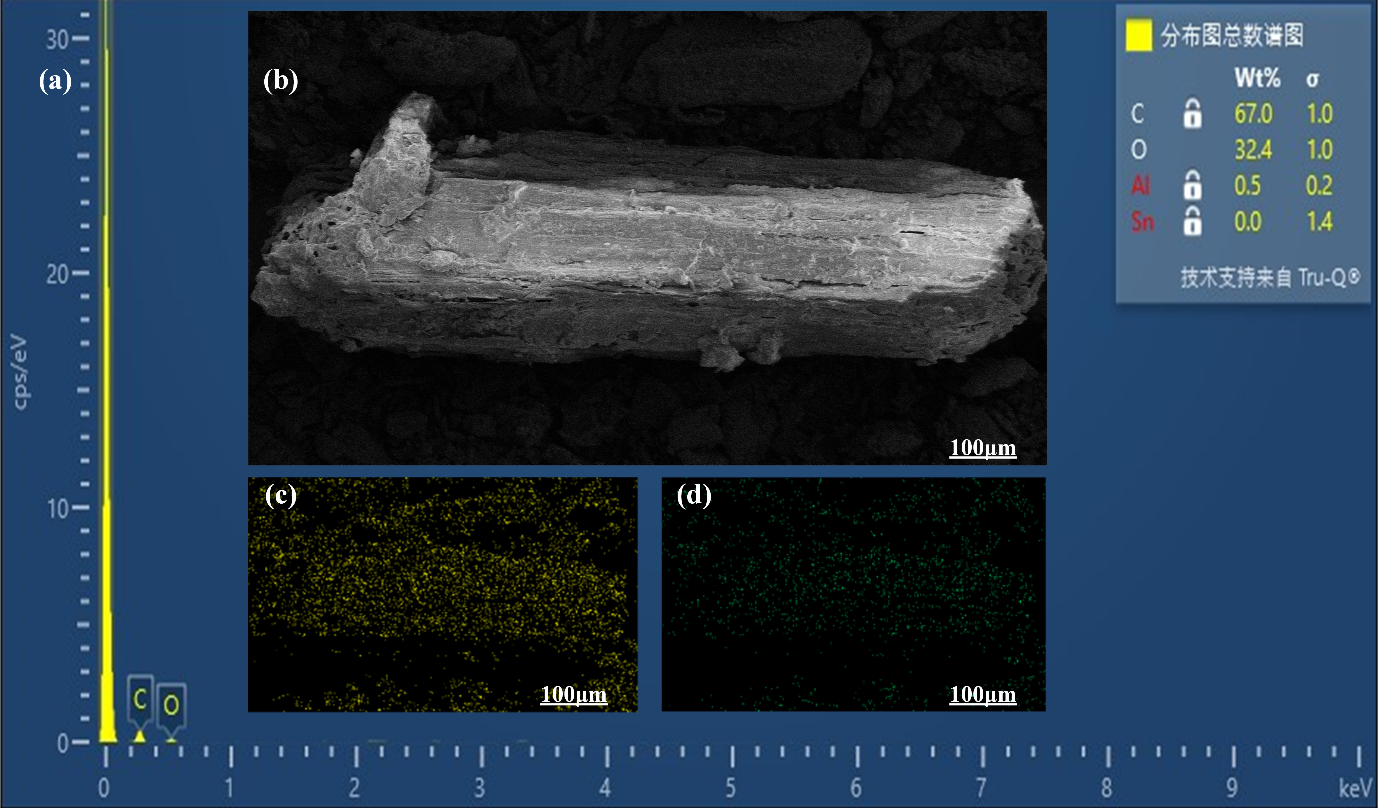


**Figure S1.** Scanning Electron Microscopy with Energy Dispersive X-ray Spectroscopy (SEM-EDX) analysis of *Wedelia trilobata* biochar, illustrating its elemental composition and distribution. (a) The overall elemental profile spectrum reveals the predominant presence of carbon (C) and oxygen (O) in the biochar. (b) A high-resolution SEM image provides a detailed view of the biochar's porous and fragmented surface structure. (c & d) Elemental mapping shows the spatial distribution of carbon (C) and oxygen (O).


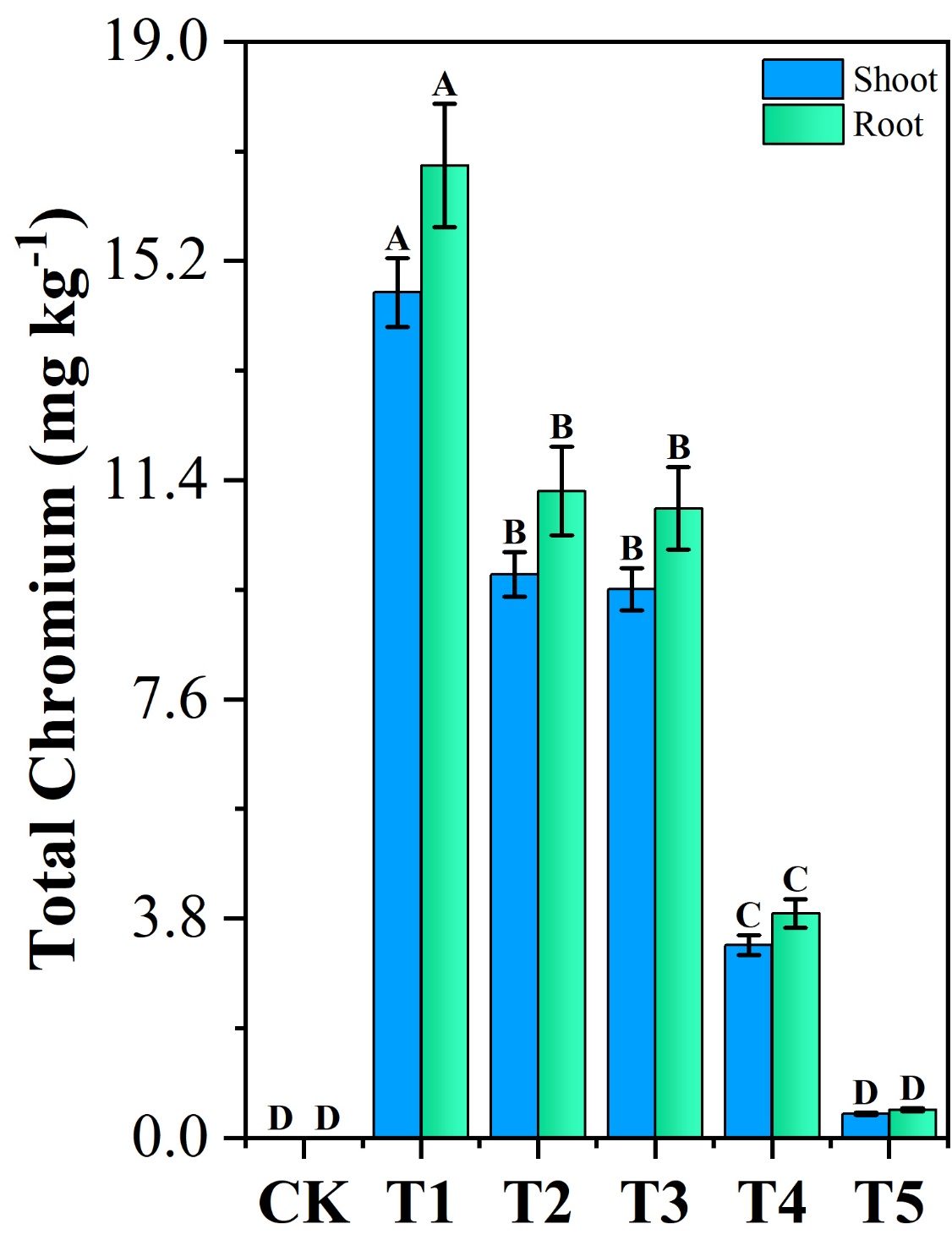


**Figure S2.** Impact of various biochar concentrations on chromium accumulation in cabbage roots and shoots. Error bars represent the standard deviation (SD) of the mean (n = 3), and different capital letters denote statistically significant differences among treatments (p < 0.05).


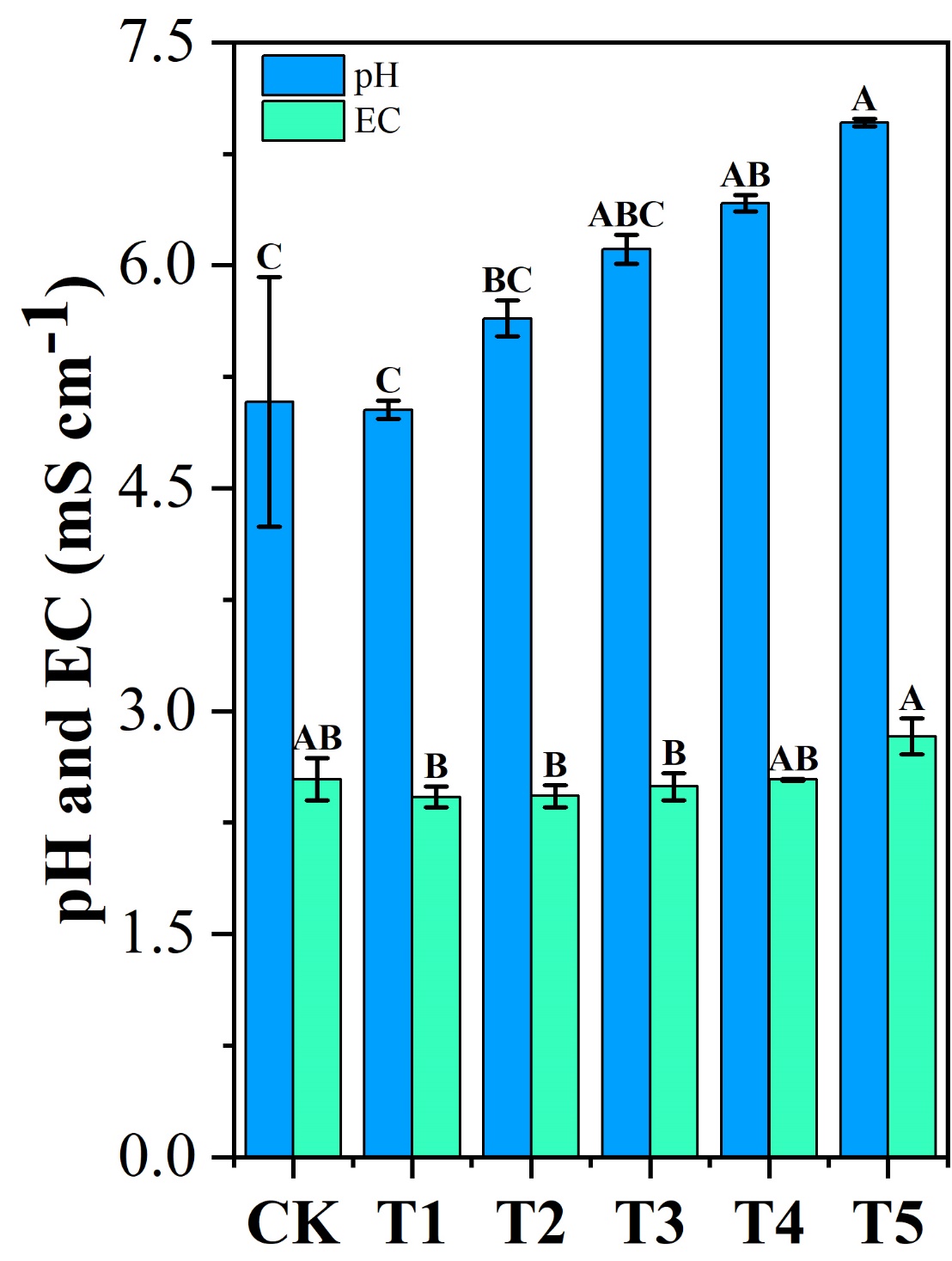


**Figure S3.** Impact of various biochar concentrations on the pH and electrical conductivity (EC) of the nutrient solution used in the hydroponic experiment. Error bars represent the standard deviation (SD) of the mean (n = 3), and different capital letters denote statistically significant differences among treatments (p < 0.05).


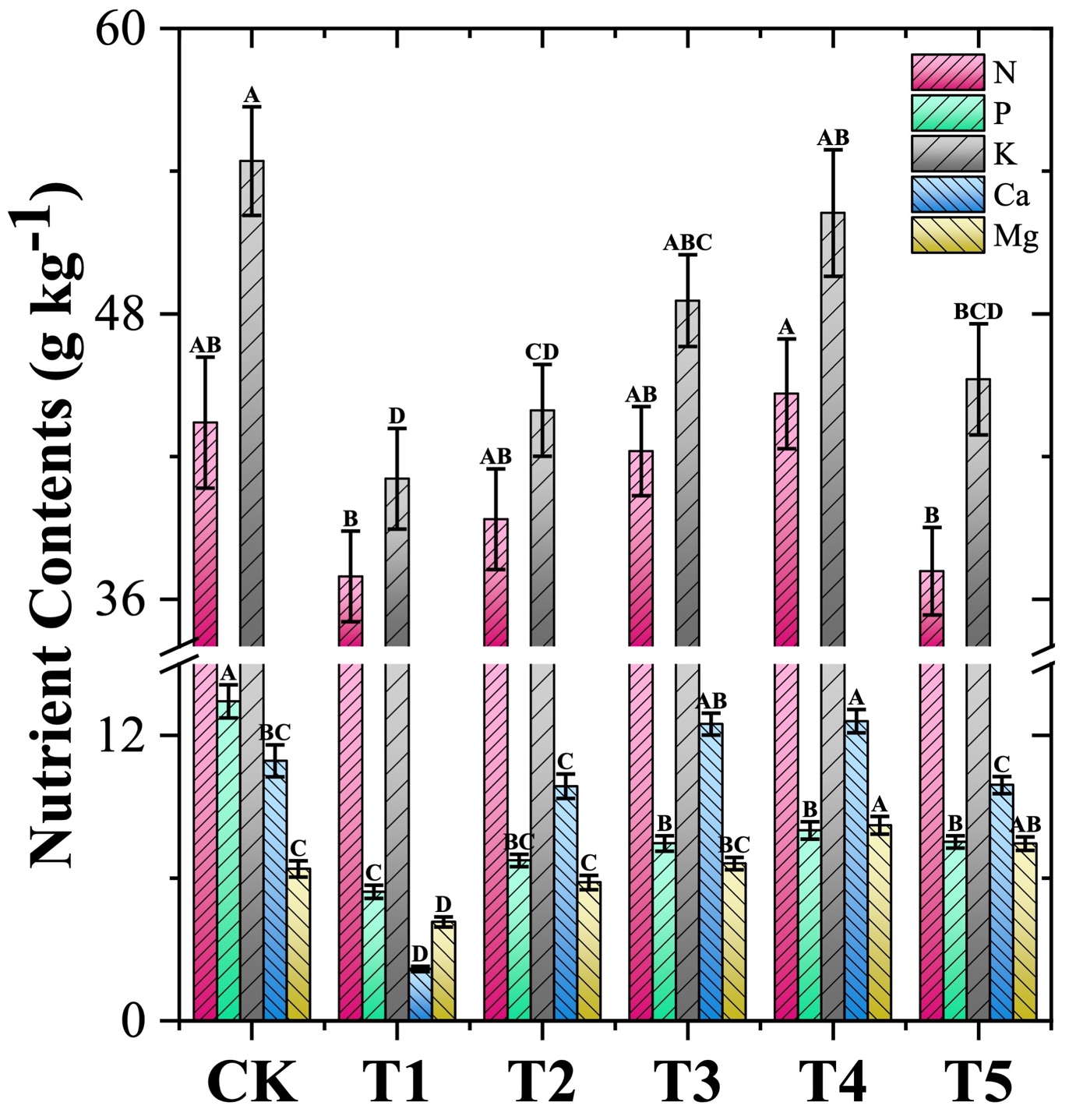


**Figure S4.** Impact of various biochar concentrations on nutrient contents in cabbage. Error bars represent the standard deviation (SD) of the mean (n = 3), and different capital letters denote statistically significant differences among treatments (p < 0.05).


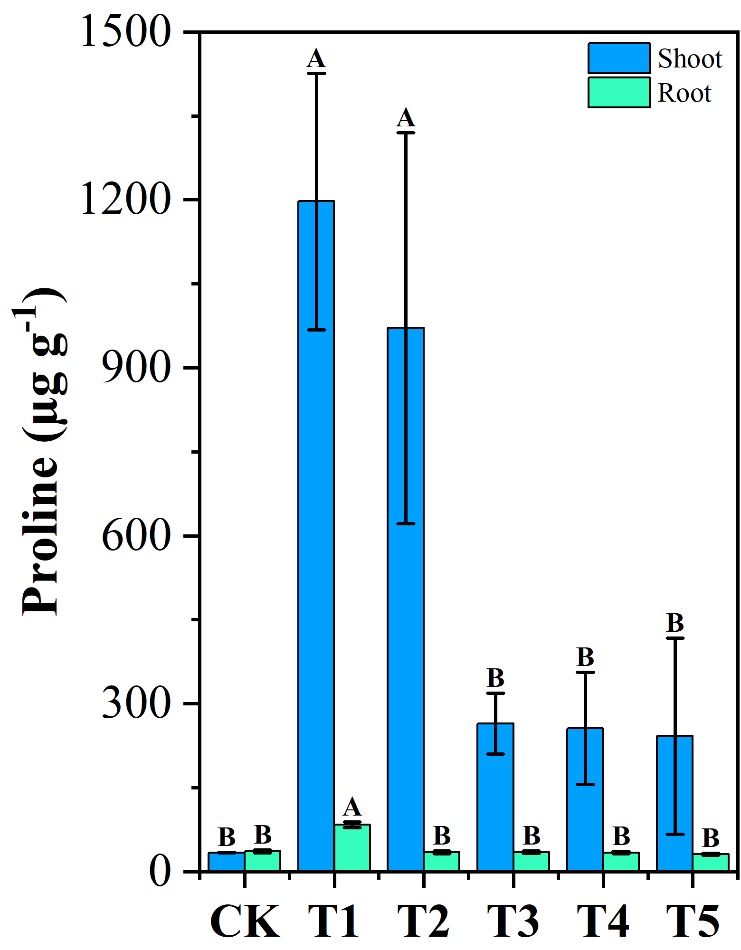


**Figure S5.** Impact of various biochar concentrations on proline levels in cabbage roots and shoots. Error bars represent the standard deviation (SD) of the mean (n = 3), and different capital letters denote statistically significant differences among treatments (p < 0.05).


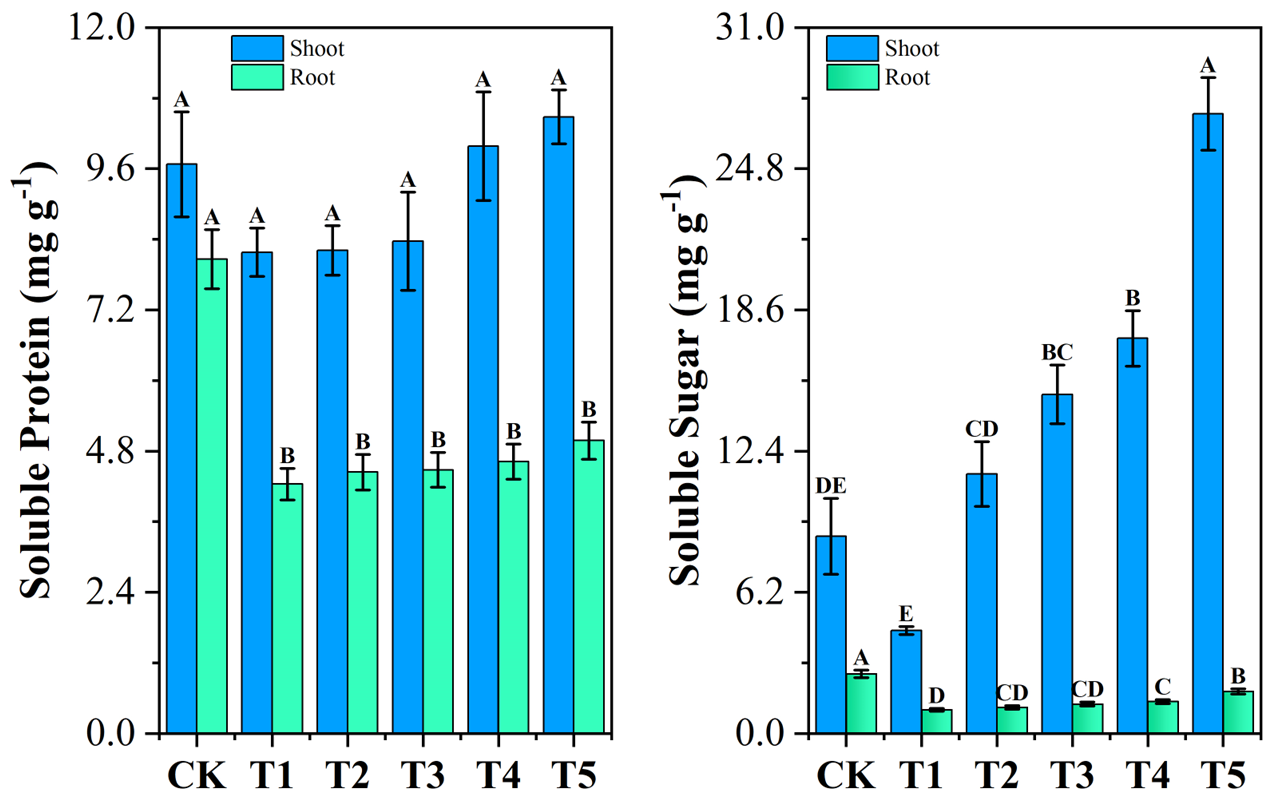


**Figure S6.** Impact of various biochar concentrations on soluble protein and sugar contents in cabbage roots and shoots. Error bars represent the standard deviation (SD) of the mean (n = 3), and different capital letters denote statistically significant differences among treatments (p < 0.05).


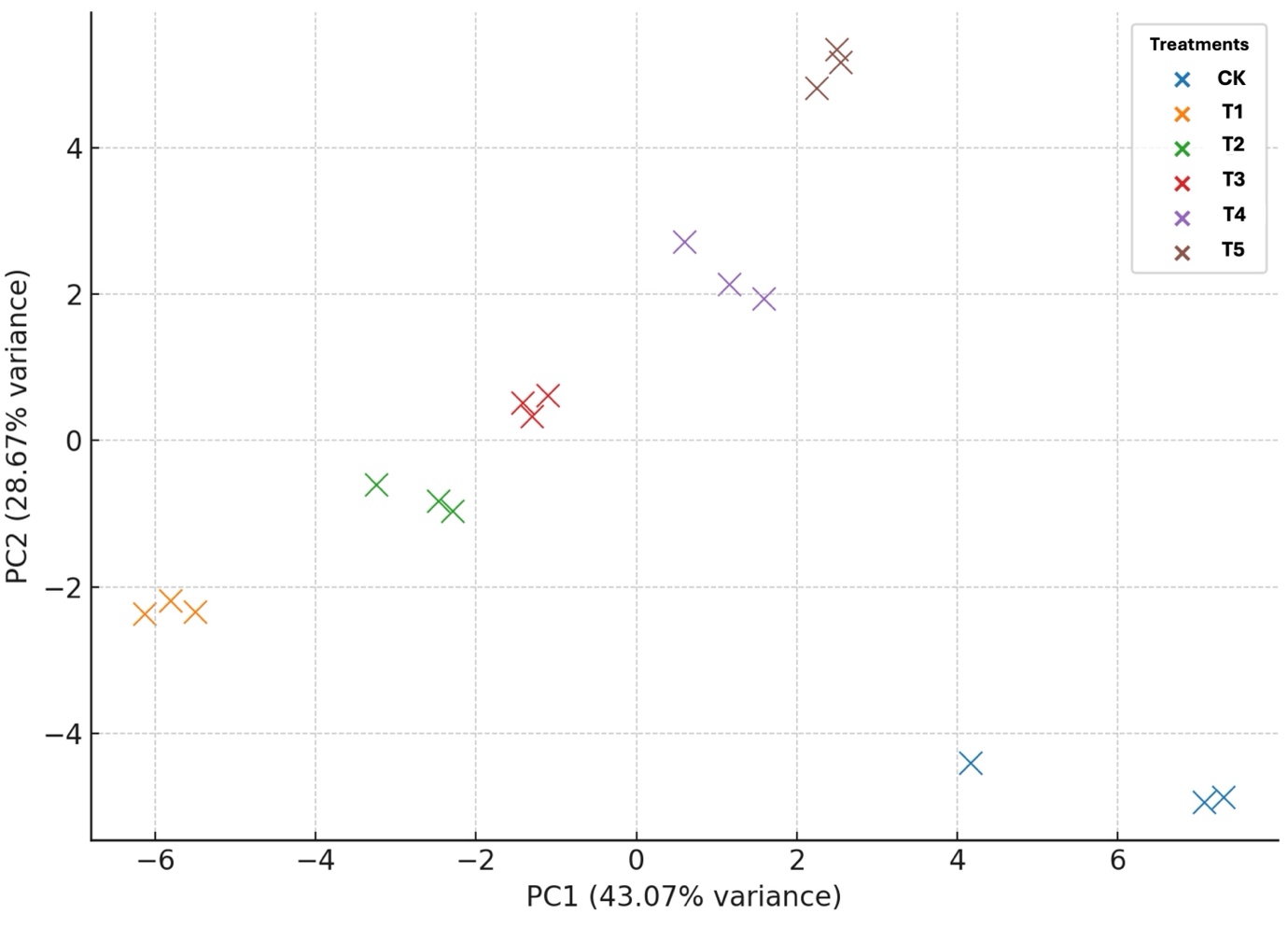


**Figure S7.** Principal Component Analysis (PCA) of plant traits in Brassica rapa under different treatments. PCA was performed to assess the multivariate variation among treatments involving chromium (Cr) stress and Wedelia trilobata-derived biochar applications. The first two principal components (PC1 and PC2) explain 43.07% and 28.67% of the total variance, respectively. Each point represents an individual replicate, colored according to treatment group, showing distinct clustering and separation among treatments.
